# Chloroplast-encoded small subunit extensions reshape the *Chlamydomonas* chlororibosome

**DOI:** 10.1038/s41477-026-02361-1

**Published:** 2026-07-27

**Authors:** Florent Waltz, Philippe A. Lehner, Philippe Van der Stappen, Lukas Kater, Stefan Pfeffer, Benjamin D. Engel

**Affiliations:** 1 Biozentrum, University of Basel, Basel, Switzerland; 2 Swiss Nanoscience Institute, University of Basel, Basel, Switzerland; 3 Friedrich Miescher Institute for Biomedical Research, Basel, Switzerland; 4 Zentrum für Molekulare Biologie der Universität Heidelberg, Heidelberg, Germany

**Keywords:** Plant molecular biology, Cryoelectron microscopy, Cryoelectron tomography

## Abstract

Chloroplast ribosomes (chlororibosomes) synthesize the core protein components of the photosynthetic apparatus, yet their structural diversity outside flowering plants remains largely unexplored. Here we combine in situ cryo-electron tomography with single-particle cryo-electron microscopy to determine the structure of the chlororibosome from the unicellular green alga *Chlamydomonas reinhardtii*. Subtomogram averaging of chlororibosomes in their native environment, resolved to ~5 Å and in distinct translational states, reveals particles both free in the stroma and flexibly tethered to thylakoid membranes. These in situ reconstructions uncover an additional ‘arm’ domain on the small subunit. High-resolution single-particle structures of isolated chlororibosomes resolved to ~2.5 Å, in states bound either to the inhibitory translation factor pY or to a nascent chain-linked P-site transfer RNA, reveal that this domain is built primarily from extensive chloroplast-encoded insertions and extensions of conserved small subunit proteins, supported by chlororibosome-specific ribosomal proteins. The arm domain is located around the mRNA entry and exit channels, suggesting a role in stabilizing the mRNA trajectory through the small subunit and organizing chloroplast polysomes. Together, these data reveal the unexpected structural variation of algal chlororibosomes and suggest that chloroplast translation has diversified substantially even among relatively closely related photosynthetic lineages.

## Abstract (web summary)

Using in situ cryo-electron tomography, researchers found a unique ‘arm’ domain on the small subunit of *Chlamydomonas* chlororibosomes. This discovery suggests that chlororibosome structure is more evolutionarily diverse than previously thought.

## Main

Chloroplasts are key organelles of photosynthetic organisms, responsible for oxygenic photosynthesis. Their origins trace back to an ancient endosymbiotic event between a eukaryotic host cell and a cyanobacterial ancestor^1^. This evolutionary merger had a profound impact on our planet, as plants and microalgae constitute the base of most food chains, producing many molecules of interest for human health as well as being major players in carbon sequestration^2–4^. Chloroplasts have also emerged as attractive ‘green factories’ for the high-level expression of heterologous proteins and resistance traits^5–7^.

Chloroplasts contain their own ribosomes, called chlororibosomes, which were inherited from cyanobacteria and are responsible for synthesizing ~75 chloroplast-encoded proteins out of ~1,000 proteins in the organelle^8–10^. Despite their central role in assembling and maintaining the photosynthetic apparatus, chlororibosomes remain much less structurally characterized than bacterial or cytosolic ribosomes. By contrast, mitochondrial ribosomes have been thoroughly studied and exhibit considerable structural and compositional diversity among eukaryotes^11–15^. Although the common consensus is that chlororibosomes are less diverse and more bacteria-like compared with mitoribosomes, our structural knowledge is solely based on chlororibosomes from the flowering plant *Spinacia oleracea*, commonly known as spinach^16–19^. However, chloroplasts are distributed across a broad phylogenetic spectrum^20^, making it likely that chlororibosomes vary in composition, architecture and interactions with regulatory and assembly factors that control chloroplast biogenesis and photosynthetic capacity^21,22^. Defining this diversity is essential for understanding how chloroplast translation is tuned to support photosynthesis.

## The *C. reinhardtii* chlororibosome harbours an additional domain on its SSU

**Fig. 1:**
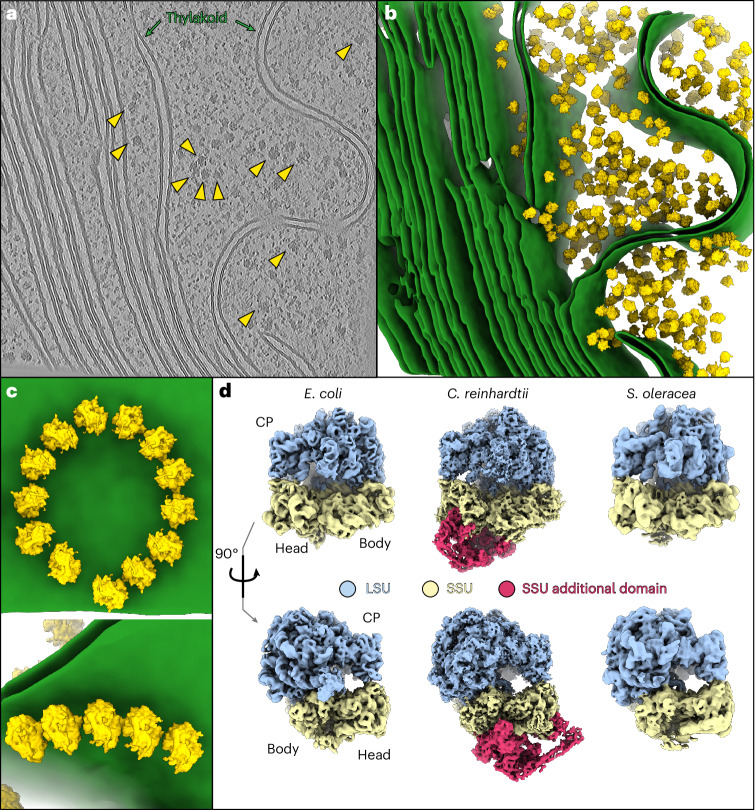
In-cell structure and organization of *Chlamydomonas* chlororibosomes. **a**, Slice through a tomogram depicting the chloroplast within a native *C. reinhardtii* cell. Yellow arrowheads point to chlororibosomes. **b**, Corresponding segmentation of the thylakoid membranes (green) with STA structures of the chlororibosomes (yellow) mapped back into their respective particle positions. **c**, Close-up views of membrane-bound chloroplast polysomes. **d**, Subtomogram average of the *C. reinhardtii* chlororibosome, highlighting the large additional ‘arm’ domain, compared with the *E. coli* bacterial ribosome and the *S. oleracea* chlororibosome. CP, central protuberance.

To probe chlororibosome diversity beyond flowering plants, we worked with *Chlamydomonas reinhardtii*, a unicellular green alga that serves as a valuable model to study chloroplast biology owing to its genetic tractability and established experimental techniques^23–25^. To image chlororibosomes in their most native state, we leveraged the publicly available *Chlamydomonas* cryo-electron tomography (cryo-ET) dataset, EMPIAR-11830^26^. We selected 129 chloroplast-containing tomograms to perform automated chlororibosome detection using template matching and subsequent subtomogram averaging (STA; Supplementary Fig. 1). Chlororibosomes were detected in the stroma, both as free and in association with polysomes (Supplementary Fig. 2), as well as bound to thylakoid membranes (Fig. 1 and Supplementary Video 1). This observation indicates that chlororibosomes are actively translating both soluble stroma proteins and membrane proteins for the photosynthetic machinery. Interestingly, we also observed large polysomes organized at the membrane, often circular, a structural feature that was first described by Chua et al. in 1976^27^ (Fig. 1c).

STA of the chlororibosomes reached ~5-Å resolution, revealing an unexpected architectural feature: a substantial additional domain on the small subunit (SSU), found on all particles. As this domain roots from the body of the ribosome and extends towards the head, we name it hereafter the SSU arm domain (Fig. 1d). Comparison with the bacterial ribosome and spinach chlororibosome structures shows that this SSU domain is absent from previously characterized chlororibosomes, suggesting that it represents a species-specific adaptation in *Chlamydomonas*. The base of the SSU arm is located around the mRNA entry and exit channels, hinting that this additional domain could be made either from new r-proteins interacting there or from extensions of these conserved r-proteins.

## Thylakoid-associated chlororibosomes are flexibly tethered yet actively translating in situ

**Fig. 2:**
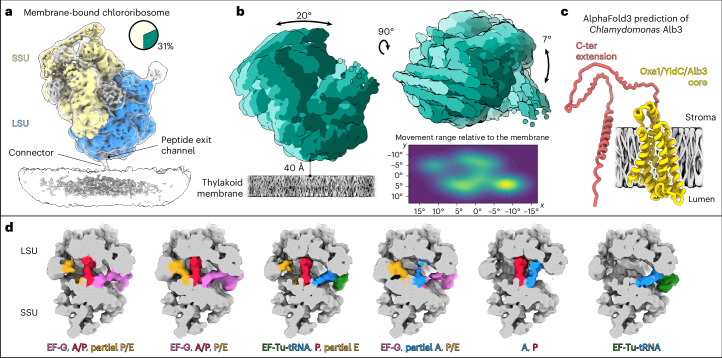
Membrane associated chlororibosomes and translation states. **a**, Subtomogram average of thylakoid-bound chlororibosomes, representing 31% of the particles. The thin proteinaceous connector between the peptide exit channel and the thylakoid membrane is indicated. **b**, Analysis of chlororibosome orientation variability relative to the membrane. The different orientations relative to the membrane are overlayed with various shades of teal, displayed from both side and top views. The heat map shows the projected orientation range of the ribosome relative to the membrane plane. **c**, AlphaFold3 prediction of *C. reinhardtii* Alb3. The conserved Oxa1/YidC/Alb3 core is shown in yellow, embedded in the thylakoid membrane, whereas the extended, flexible C-terminal region is shown in coral and projects into the stroma. **d**, The distinct translation states resolved from the complete chlororibosome dataset. Subtomogram averages are shown for the elongation factor and tRNA-bound states. tRNAs and translation factors are coloured and annotated as indicated.

Subtomogram classification revealed that only a fraction of chlororibosomes are positioned adjacent to thylakoid membranes (~31%), whereas the majority remain free in the stroma (Fig. 2a). Thylakoid-associated ribosomes are relatively distant from the membrane surface compared with cytosolic or mitochondrial ribosomes (~40 Å from the peptide exit channel to the membrane). On average, only a weak, thin density bridges the ribosomal exit region (near ribosomal RNA (rRNA) helix H59) with the membrane (Supplementary Fig. 3a). This suggests that the tether, probably comprising the nascent polypeptide and/or components of a thylakoid protein translocase, is highly flexible or compositionally heterogeneous, allowing substantial lateral and angular motion of the ribosome relative to the membrane (Fig. 2b). These observations support a model in which chlororibosomes engage the thylakoid translocon in a dynamic manner, in contrast to cytosolic ER-bound ribosomes and mitochondrial ribosomes, which are more rigidly docked (Supplementary Fig. 3a). This is confirmed by the range of chlororibosome orientations observed relative to the thylakoid membrane, spanning 20° in the most extreme case (Fig. 2b). On the ribosome side, the area surrounding the peptide exit channel is particularly bacterial-like and does not show specific domains or remodelling that would support a specialized translocon interaction^28^ (Supplementary Fig. 3b). Consistently, bL23c is more similar to its bacterial counterpart than its spinach counterpart, arguing against a possible alternative exit channel in *Chlamydomonas* chlororibosomes^17^.

In the flowering plant *Arabidopsis*, the protein Alb3, belonging to Oxa1/YidC/Alb3 protein family and also involved in mitoribosome membrane tethering^28–30^, has been shown to be the possible factor involved in co-translational protein insertion in the chloroplast^31,32^. The *Chlamydomonas* homologue (Uniprot: Q8LKI3) has a disordered and extended C-terminal region that is a strong candidate for mediating ribosome binding, consistent with the flexible connector observed here (Fig. 2c). Alb3 was also previously identified by mass-spectrometry as the sole translocase co-immunoprecipitated with *Chlamydomonas* chlororibosomes^33^. However, at ~8-Å resolution for the membrane-bound subclass, the map did not permit unambiguous visualization of density corresponding to this putative C-terminal segment bound to the ribosome.

To assess whether the chlororibosomes are translationally active, we performed subtomogram classification and averaging focused on the intersubunit space. This analysis resolved multiple transfer RNA (tRNA) occupancy states in situ, including configurations and translation factors characteristic of the elongation cycle (Fig. 2d). Although these structural snapshots are consistent with active translation, we acknowledge that ribosomes in these configurations may also represent complexes stalled during elongation. Moreover, the EF-Tu-tRNA state could also be compatible with the presence of the pY hibernation factor. Both free and thylakoid-associated chlororibosomes populate these states, suggesting that the membrane-bound complexes are actively translating and engaged in co-translational insertion and/or processing of nascent thylakoid proteins. In addition, both the soluble and thylakoid-bound ribosomes had the arm domain.

The discovery of the SSU arm domain and its presence on translationally active chlororibosomes raised the question of its molecular composition. However, at the resolution of our in situ average, we could not unambiguously assign specific polypeptides to this domain.

## Complete composition of the *Chlamydomonas* chlororibosome solved by single-particle cryo-EM

**Fig. 3:**
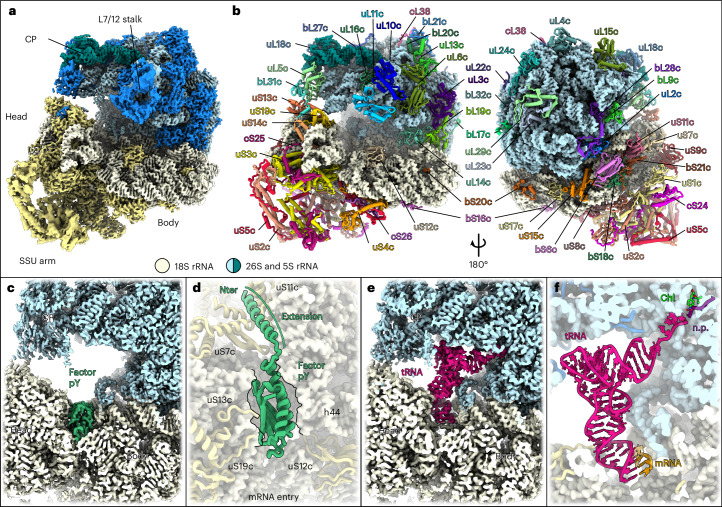
Single-particle structure of isolated chlororibosomes. **a**, Consensus composite single-particle reconstruction of the *C. reinhardtii* chlororibosome, with components of the SSU shown in beige and components of the LSU shown in blue shades. **b**, Resulting atomic model, with proteins shown in cartoon representation and rRNA shown as surface. All proteins are annotated. **c**,**d**, Zoomed views of the pY-bound chloribosome: density (**c**) and corresponding model (**d**) with pY in green. **d**, pY is shown from the LSU, with the N-terminal extension and the surrounding r-proteins annotated. **e**,**f**, Zoomed views of a bound tRNA: density (**e**) and corresponding model (**f**) with tRNA in magenta, mRNA in orange and chloramphenicol (Chl) near the nascent polypeptide (n.p.). CP, central protuberance.

To clarify the identity of the arm domain and obtain a complete model of the *Chlamydomonas* chlororibosome, we proceeded to solve its structure using cryo-electron microscopy (cryo-EM) single-particle analysis (SPA). Chlororibosomes were purified from whole cell lysate using sucrose density gradient centrifugation. Two datasets were collected, one of which was treated with chloramphenicol to stall translation. During image processing (Supplementary Fig. 4), we observed that the additional arm domain density was only resolved in a subset of particles (~24%). Considering that the domain is always present in situ, its low occupancy in SPA is most likely a result of the purification. Refinement of all particles yielded a 2.30-Å consensus reconstruction, whereas restricting the analysis to the subset showing the arm domain resulted in a 2.52-Å map that was further improved by focused refinements on different regions of the chlororibosome (Fig. 3a and Supplementary Figs. 4 and 5).

This high-resolution SPA map allowed the unambiguous identification of a total of 55 proteins, 31 in the large subunit (LSU) and 24 in the SSU (Fig. 3b), which we consider to be the complete set of core r-proteins of this chlororibosome (see table of proteins in Supplementary Table 2 for more details). Compared with a bacterial ribosome such as *Escherichia* *coli*, the *Chlamydomonas* chlororibosome possess a complete set of r-proteins except for the absence of bL25 and uL30 from the LSU, which was also previously described in Angiosperm chlororibosome structures^16–19^. On the LSU, we find one chloroplast-specific r-protein, cL38/PSPR6, which is conserved with flowering plants, whereas cL37/PSPR5 is not present (Supplementary Fig. 6).

In flowering plants, the chloroplast LSU rRNA is composed of the 5S, 4.5S and 23S rRNAs; the latter is further fragmented post-transcriptionally into three pieces termed A, B and C^16^. In *Chlamydomonas*, the rRNAs are differentially split (Supplementary Fig. 7). There is a total of four rRNA pieces: the 5S, 7S, 3S and 23S, which are encoded as such in the chloroplast genome^9^. The 7S rRNA forms most of domain I, except for helices H19 and H20, which are formed by the 3S rRNA. Helix H18, which would normally connect domain I to helices H19 and H20, is absent. This leaves the small 3S rRNA fragment stabilized only by r-proteins uL4c and uL24c (Supplementary Fig. 7b). This arrangement helps preserve a bacterial-like architecture in the region surrounding the peptide exit channel. By contrast, the SSU rRNA does not show notable changes from the bacterial organization.

Our cryo-EM map resolution enabled us to visualize rRNA modifications, which are hallmarks of subunit maturation towards a translationally competent ribosome and were not resolved in previous chlororibosome structures. In total, we identified 18 rRNA modifications, 7 in the SSU and 11 in the LSU (Supplementary Fig. 8), all at positions conserved with bacteria. Additional modifications, such as pseudouridine, may be present but could not be resolved, as isomerization modifications generally require ~2.0-Å resolution for unambiguous identification^34^. Still, this agrees with the conclusion that the core of the chlororibosome is fundamentally bacterial.

During the analysis, we found that the majority of chlororibosomes were bound to the translation factor pY (also known as PSRP1) (Fig. 3c). This factor was previously described bound to the flowering plant chlororibosome and is involved in light- and temperature-dependent control of protein synthesis^16–19,35–38^. As described in these studies, we observe pY bound in the mRNA channel of the SSU, where it contacts the 16S rRNA and blocks tRNA binding at both the A and P sites, thereby inhibiting translation (Fig. 3d). pY is present in all previously published chlororibosome structures^16–19^, most likely because it associates with chlororibosomes early during purification, when centrifugation is performed in the dark and subsequent steps at 4 °C. Compared with land-plant pY, the *Chlamydomonas* factor has a similar core that binds the mRNA channel in an equivalent manner, but it harbours an additional C-terminal extension that projects into the mRNA exit channel (Fig. 3d). To reduce pY binding and obtain a structure of the chlororibosome stalled in a translating state, we also purified them from cells preincubated in the presence of chloramphenicol^39^. Thereby, we obtained a stalled chlororibosome structure at 2.84-Å resolution with a P-site tRNA, a segment of mRNA clearly resolved in the mRNA channel, bound chloramphenicol and the nascent polypeptide chain (Fig. 3e,f). Although the mode of interaction of the tRNA, mRNA and chloramphenicol with the chlororibosome is identical compared with bacteria, this structure provides a stalled translating chlororibosome that we used to investigate the role of the SSU extension (see below).

## The arm domain is primarily formed by large extensions in conserved SSU r-proteins

**Fig. 4:**
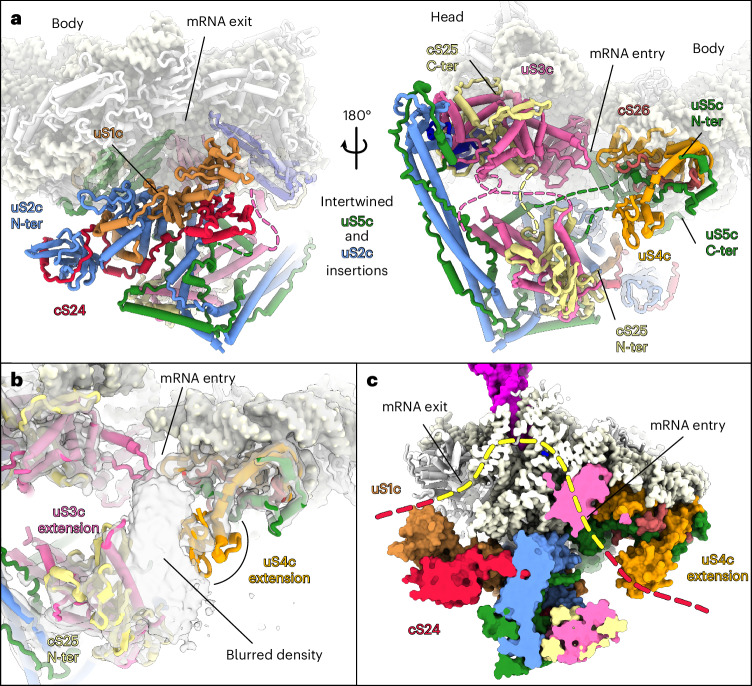
The SSU arm domain extends the entry and exit of the mRNA channel. **a**, Overall architecture of the SSU arm. Two views related by a 180° rotation show the arrangement of arm components from the mRNA exit and entry. Proteins are shown in cartoon representation. **b**, A close-up of the mRNA entry site. The model is overlayed with the map of the tRNA class. A blurred density is sandwiched between the uS4c extension and the cS25 N-terminus, in proximity to the uS3c extension, consistent with an averaged segment of mRNA. **c**, Cut view of the SSU highlighting the prolonged mRNA path shaped by the arm domain. The canonical path is indicated in yellow (dashed), whereas the prolonged path observed here is shown in red (dashed). The mRNA entry is stabilized mainly by the uS4c extension and the exit by uS1c, which itself is stabilized by cS24.

The most pronounced differences at the protein and structural levels are found in the SSU. Relative to bacterial and plant chloroplast ribosomes, the SSU contains a nearly complete set of canonical r-proteins, with the notable exception of bTHX, which is absent here, although present in the *Chlamydomonas* mitochondrial ribosome^29^. In addition, chloroplast-specific r-proteins cS22 and cS23 (PSRP2 and PSRP3), which in flowering plants localize to the SSU foot^16^, are entirely missing (Supplementary Fig. 6). Together, these features render the SSU strongly bacterial-like, apart from the extended SSU arm domain that projects from this conserved core (Fig. 4).

The SSU arm is positioned on the solvent-exposed side of the SSU, anchored around the mRNA entry and exit channels (SSU back) of the SSU body, from which a long, straight segment extends towards the SSU head. It is formed by extensions and insertions in the r-proteins uS2c, uS3c, uS4c and uS5c, together with three chloroplast-specific r-proteins, PSRP3, PSRP7 and the previously uncharacterized PSRP8, which are not present in flowering-plant chlororibosomes (Supplementary Fig. 9). Following ribosomal protein nomenclature^40^, and because they are core components of the *Chlamydomonas* chlororibosome, we refer to these additional proteins as cS24, cS25 and cS26, respectively. cS24/PSRP3 is located at the mRNA exit channel on the back of the SSU, where it stabilizes the interface between the conserved cores of bS1c and uS2c (Supplementary Fig. 6). cS25/PSRP7 is the largest (560 amino acids) of the chlororibosome-specific proteins. Its C-ter part is positioned on the beak region of the SSU head, where it is intertwined with the extensions of uS3c and a portion of uS10c, whereas its N-ter part extends to the core of the SSU arm domain. On the body, cS26/PSRP8 functionally replaces the C-ter portion of uS4c, which in *Chlamydomonas* has an extension that forms a specific domain. There, cS26/PSRP8 interacts with the core parts of uS4c and uS5c. Together, these chloroplast-specific proteins appear to stabilize regions of the chlororibosome that harbour extensive r-protein extensions.

Most of the SSU arm domain, however, is contributed by extensions in uS2c, uS3c, uS4c and uS5c, which was previously hinted by early proteomic studies^33,41,42^. Relative to their bacterial counterparts, uS2c, uS3c, uS4c and uS5c are extended by 666, 497, 97 and 512 residues, respectively (Supplementary Fig. 10). The relatively small C-terminal extension of uS4c folds into a globular domain near the mRNA entry channel. By contrast, uS2c is the protein with the largest extension. On its N-ter, the extension forms three S1-like β-barrel domains that run along the SSU body in proximity to uS8c. Only the third (most C-terminal) S1-like domain could be fully modelled, stabilized by interactions with cS24/PSRP3 (Supplementary Fig. 11). The first two S1-like domains are poorly resolved in SPA reconstructions but are clearly visible in the native STA map (Supplementary Fig. 11c). The insertion of uS2c (440–795 amino acids) consists mainly of alpha helices that form a coiled-coil extending away from the SSU back, where it intertwines by charge complementarity with the unstructured extension of uS5c (Supplementary Fig. 10a,c). The intertwined uS2c/uS5c arm reaches towards the SSU head to contact part of the extension made by uS3c and is further stabilized by the N-terminal segment of uS10c. As only a few residues participate in this interface, the interaction is probably weak, and the entire uS2c/uS5c arm may readily unlatch. This would explain why the native conformation was retained in only a subset of chlororibosomes visualized by SPA; the arm domain probably unlatched during isolation and was therefore too flexible to be resolved in the average. Finally, uS3c extensions enlarge the SSU beak region but also project at the base of the intertwined uS2c/uS5c segment. There, it is made of several alpha helices that interweave with the N-ter part of cS25/PSRP7 to form a platform-like structure (Fig. 4). Using Foldseek^43^, we analysed the conservation of the SSU arm. The analysis worked best on uS2c and uS3c, which have the most defined folds, and shows that this domain is restricted to Chlorophyceae (Supplementary Fig. 12).

Looking at the SPA reconstructions of chlororibosomes bound to P-site tRNA, we observed a blurred density near the SSU arm domain, positioned between the uS4c extension and the intertwined cS25 and uS3c extensions (Fig. 4b). This density most likely corresponds to averaged mRNAs stalled on the ribosome, which are expected in the tRNA class. The uS4c and uS3c extensions positioned there are overall positively charged (Supplementary Fig. 10b), which is compatible with a role in mRNA stabilization. These observations suggest that the uS4c extension and the platform made of cS25 and uS3c extensions help stabilize and/or thread the mRNA at the mRNA entry channel. On the other side, at the mRNA exit, the protrusion formed by cS24/PSRP3, uS2c and bS1c (Fig. 4a) also points towards stabilization of the mRNA at the exit channel (Fig. 4 and Supplementary Fig. 11). Together, the r-protein extensions and small proteins of the arm domain appear to prolong the mRNA path, at both the mRNA entry side (uS4c, uS3c and cS25) and the mRNA exit side (cS24/PSRP3, uS2c and bS1c) of the chlororibosome SSU (Fig. 4c).

In summary, our SPA and in situ cryo-ET reveal that *Chlamydomonas* chlororibosomes adopt a dynamic mode of membrane engagement and harbour an accessory arm domain on the SSU, an unexpected discovery that refutes the previous assumption that all chlororibosomes are structurally similar. In *Chlamydomonas*, membrane-bound chlororibosomes are only loosely tethered to thylakoids via a thin connector that is consistent with a flexible Alb3–nascent chain interaction, allowing substantial orientational freedom rather than rigid docking at the translocon. This structural plasticity could represent an evolutionary strategy to accommodate the chloroplast’s large soluble proteome by allowing ribosomes to transition dynamically between membrane-bound and soluble states. Such flexibility could also facilitate the rapid recruitment and release of chlororibosomes, for example in photosystem repair, enabling efficient adaptation to fluctuating light environments. The SSU arm domain emerges as a noticeable architectural innovation: it is built predominantly from chloroplast-encoded extensions and insertions in canonical SSU r-proteins, together with three chloroplast-specific r-proteins. Its position decorates the mRNA entry and exit channels (Fig. 4c), suggesting roles in stabilizing the mRNA and possibly organizing chloroplast polysomes. Moreover, the long arm structure projecting from the back towards the head might play a role in recruiting regulatory factors, although the precise binding partners remain unknown. Supporting this hypothesis, several gene-specific *trans*-acting proteins have been described to be involved in mRNA stabilization and required for efficient translation in the *Chlamydomonas* chloroplast^44–46^. Given that *Chlamydomonas* is relatively close to land plants in the green lineage, our findings imply that chlororibosomes are probably more structurally and functionally diverse than previously appreciated, with potentially even greater elaboration in secondary plastid-bearing algae that dominate marine phytoplankton and remain to be explored.

## Methods

### In situ cryo-ET

#### Data acquisition and preprocessing

All data analysis was performed on the cryo-ET dataset deposited under EMPIAR-11830^26^. In brief, tilt-series data were collected using a Titan Krios G4 transmission electron microscope operating at 300 kV equipped with a Selectris X energy filter with a slit set to 10 eV and a Falcon 4i direct electron detector (Thermo Fisher Scientific) recording dose-fractionated movies in EER format. A dose-symmetric tilt scheme using TEM Tomography 5 software (Thermo Fisher Scientific) was used in the acquisition with a tilt span of ±60°, covered by 2° or 3° steps starting at a 10° pre-tilt. Target focus was set for each tilt-series in a range of −1.5 µm to −3.5 µm in steps of 0.25 µm. The microscope was set to a magnification corresponding to a pixel size of 1.96 Å at the specimen level and a nominal dose of 3.5 e^− ^Å^−2^ per tilt image.

#### STA

In total, 129 chloroplast tomograms (Supplementary Table 3) from the *Chlamydomonas* dataset^26^ were selected on the basis of overall quality and the abundance of chloroplast ribosomes. Data processing followed the workflow described in the TomoGuide^47^ using a postacquisition refined pixel size of 1.91 Å (refs. ^26,48^). See Supplementary Fig. 1 for the processing workflow. Using AreTomo3^49^, raw EER movies were automatically motion-corrected, their CTF estimated, tilt-series-aligned and CTF-corrected tomograms reconstructed. Denoising was performed with Icecream on tomogram pairs reconstructed from odd or even raw frames respectively^50^.

For particle picking, three-dimensional (3D) template matching was carried out on bin4 CTF-corrected tomograms (7.64 Å/pixel) with 6° angular sampling in pyTOM-match-pick^51^, using a chloroplast ribosome reference obtained from SPA and low-pass-filtered to bin4 Nyquist (15.28 Å). For each tomogram, the number of extracted positions were automatically determined by pytom, and the threshold was reduced by 5% to maximize the number of true positives, yielding an initial set of 20,883 picks. Picks were then imported in RELION5^52^, and the particles were cleaned by several rounds of 3D classification without alignment using bin4 subtomograms, to clean out obvious false positives (membranes and high signal contaminants). At this step, the 14,415 remaining particles were 3D-refined and reached bin4 Nyquist (15.28 Å).

From there, a bin1 reference (8.87 Å) was generated for CTF refinement and Bayesian polishing, improving the overall resolution to 6.93 Å. Particles were reextracted at bin2, 3D-refined to 7.64 Å and cleaned by an additional round of 3D classification, resulting in 13,245 particles. A new bin1 reference (6.79 Å) was used for a second round of CTF refinement and Bayesian polishing, yielding a 6.70-Å map. Finally, particles were reextracted at bin1, refined to 6.39 Å, and followed by a final round of CTF refinement and Bayesian polishing, which resulted in a 6.11-Å reconstruction. Focused refinements using masks on the LSU, the SSU and the SSU extension yielded maps at 5.76 Å, 6.36 Å and 8.48 Å, respectively.

In addition, particles were reextracted at bin3 to classify for tRNA states and membrane-bound ribosomes. For membrane-bound ribosomes, 3D classification was performed without alignment in two successive rounds using a mask specifically covering the membrane area of the particles. This yielded a subset of 4,160 particles, which were used to generate the final 9.29-Å resolution reconstruction. For the tRNA states analysis, a similar approach was used using a mask covering the tRNA region. Particles were two times classified using a high *T* parameter (*T* = 10), which resulted in six distinct states.

#### Polysome analysis

Ribosome particle positions and orientations were extracted from the RELION5 particle list produced by STA. Candidate neighbours were identified per tomogram using a Euclidian KDTree search. For each candidate pair, the distance and their relative orientation was computed. The relative orientation was determined as the minimum SO(3) rotation angle required to align the two particle orientations. Candidate polysomes were accepted if their centre-to-centre distance fell within the expected range (200–320 nm) and if their relative orientation difference was below the chosen angular threshold (60°). The SO(3) orientation difference between two ribosome particles was defined as the magnitude of their relative rotation. For two particle orientations *R*_*1*_ and *R*_2_, the relative rotation is

$${R}_{{rel}}={R}_{1}^{-1}{R}_{2}$$

The corresponding angular difference is

$$\theta ={\cos }^{-1}\left(\frac{\mathrm{tr}\left({R}_{\mathrm{rel}}\right)-1}{2}\right),$$

where θ is the smallest 3D rotation angle required to align particle 1 to particle 2. Here, *R*_*1*_ and *R*_*2*_ denote the 3D rotation matrices generated from the RELION Euler angles of the two particles. For each particle *i*, the rotation matrix ${R}_{i}$ was computed as ${\mathrm{Rot}}_{i}\,$, ${\mathrm{Tilt}}_{i}$ and$\,{\mathrm{Psi}}_{i}$ using the ZYZ convention

$${R}_{i}={R}_{z}\left({\mathrm{Rot}}_{i}\right){R}_{y}\left({\mathrm{Tilt}}_{i}\right){R}_{z}\left({\mathrm{Psi}}_{i}\right)$$

applied in sequence. The individual rotation matrices are

$${R}_{z}\left({\rm\alpha }\right)=\left(\begin{array}{ccc}\cos {\rm\alpha } & -\sin {\rm\alpha } & 0\\ \sin {\rm\alpha } & \cos {\rm\alpha } & 0\\ 0 & 0 & 1\end{array}\right),$$

$${R}_{y}\left(\beta \right)=\left(\begin{array}{ccc}\cos \beta & 0 & \sin \beta \\ 0 & 1 & 0\\ -\sin \beta & 0 & \cos \beta \end{array}\right).$$

From this, polysomes were represented as graph components, where each ribosome particle is a node and each accepted neighbour distance an edge. Edges were selected hierarchically by shorter distance and lower orientation mismatch (higher orientation similarity), while enforcing a maximum graph degree of two for every ribosome so that no particle could be connected to more than two neighbours. Connected components were accepted as polysomes if they contained at least a defined minimum number of ribosomes (3 or 4). Detected polysomes were visualized in two dimensions by projecting all particle coordinates in each tomogram onto their first two principal components as well as in 3D, showing the orientation of each ribosome. The chain length distribution was calculated as the number of ribosomes per detected polysome. The full script and analysis are available via GitHub at https://github.com/Phaips/ChloroRibo.

### Ribosome-membrane orientation analysis

From the membrane-bound ribosomes, particles were further 3D classified without alignment using a mask specifically covering the membrane area. Using two different *T* parameters (*T* = 2 or 4), eight different membrane orientation classes were obtained. From there, orientations of the ribosomes relative to their membranes were quantified by finding the 3D normal vector of the plane fitted to the membrane density. Percentile thresholding of the voxel intensity in an annular region was used for membrane identification. The normal vector was determined through principal components of the two-dimensional (2D) in-plane orientation and interpolated by linear regression across the *Z*-slices. Angular deviations from the mean normal orientation were projected onto a 2D tangent plane and visualized as a kernel density map. The full script is available via GitHub at https://github.com/Phaips/ChloroRibo.

### Single-particle Cryo-EM

#### Purification of choloroplastic ribosomes

Chloroplast ribosomes were purified from 2 l of *Chlamydomonas* culture (WT2.2 strain), grown under continuous light in TAP media. Cells were collected by centrifugation at 2,000*g* for 5 min at room temperature. They were then resuspended in 30 ml lysis buffer (20 mM HEPES–KOH—pH7.6, 75 mM KCl, 15 mM MgCl_2_, 1.5 mM DTT, C0mplete EDTA-Free), and cells were ruptured using a French Press by two passes at 900 Psi pressure; 1% (final concentration) of Triton X-100 was added to the lysed cells. The solution was transferred to a 40-ml glass Dounce potter and homogenized by ten strokes to ensure the complete disruption of the chloroplast and solubilization of the membranes. Cell debris were removed by two rounds of centrifugation at 500*g* for 5 min. From there, ribosome purification followed the same procedure as previously described^53^. The supernatant was loaded onto a 40% sucrose cushion in monosome buffer (same as lysis buffer but without Triton X-100) and centrifuged at 235,000*g* for 3 h at 4 °C. The crude ribosome pellet was resuspended in monosome buffer and loaded onto a 10–30% sucrose gradient prepared using the BioComp gradient master 108 and run for 16 h at 65,000 g in SW41 rotor. Fractions corresponding to chlororibosomes were collected using a BioComp piston gradient fractionator, pelleted and resuspended in monosome buffer. For the chloramphenicol stalled ribosomes, cells were incubated with chloramphenicol for 1 h at a concentration of 500 µg ml^−1^ before cell lysis, and 100 µg ml^−1^ was kept throughout the entire purification processes.

#### Grid preparation

In total, 4 µl of chloroplast ribosomes at a concentration of 3 µg µl^−1^ of proteins (BSA equivalent measurement on Nanodrop) were applied onto a Quantifoil R2/1 300-mesh holey carbon grid coated with 2 nm continuous carbon film that were glow-discharged at 5 W for 20 s in a Gatan glow discharger. The sample was plunge frozen in liquid ethane with a Vitrobot Mark IV system (temperature 4 °C, humidity 100% and blot force 5) with a wait time of 25 s before blotting for 2.5 s.

#### Data collection

Data collection was performed using a 200 kV Glacios and a 300 kV Titan Krios electron microscope (Thermo Fisher Scientific) both equipped with Falcon4 camera using EPU (Thermo Fisher Scientific) for automated data acquisition. Data were collected at a nominal underfocus of −0.8 to −2.0 µm at a magnification of 120,000× for the Glacios and 75,000× for the Krios, both yielding a pixel size of 0.84 Å. For the 200-kV dataset, 15,628 micrographs were recorded as a .tif movie stack; each movie stack was fractionated into 50 frames for a total exposure corresponding to an electron dose of 49 e^−^ Å^−2^. For the 300-kV dataset, 20,779 micrographs were recorded as a .tif movie stack; each movie stack was fractionated into 51 frames for a total exposure corresponding to an electron dose of 50 e^−^ Å^−2^.

#### Single-particle cryo-EM data processing

For both datasets, all single-particle processing was carried out in cryoSPARC^54^, and the workflows are summarized in Supplementary Figs. 3 and 4. The 200-kV and 300-kV datasets were preprocessed, and particles were picked, classified and refined independently; only at the final stage were selected particles from the two datasets merged. After motion correction and CTF estimation, 15,491 micrographs (dataset 1, 200 kV) and 19,406 micrographs (dataset 2, 300 kV) were retained.

For the 200-kV dataset, particles were picked using Template Picker, extracted in a 648-pixel box downsampled to 256 pixels (2.126 Å per pixel) and cleaned by two rounds of 2D classification, yielding 936,493 particles. Subsequent 3D cleaning using heterogeneous refinement and 3D classification produced 491,158 good particles. For the 300-kV dataset, an equivalent pipeline yielded 456,710 good particles after 3D classification. Chlororibosomes were then classified on the basis of the presence of the SSU extension using a mask encompassing the entire SSU and the extension, using a combination of 3D classification and 3D variability analysis. This yielded 129,415 particles with the SSU extension in the 200-kV dataset and 97,236 particles with the SSU extension in the 300-kV dataset.

Particles were reextracted for high-resolution refinement in a 648-pixel box downsampled to 512 pixels (1.063 Å per pixel). For the 200-kV dataset, the SSU extension class reached 3.10 Å after global refinement, improved to 2.76 Å after combined global and local CTF refinement and finally to 2.60 Å after reference-based motion correction followed by another round of global and local CTF refinement. For the 300-kV dataset, the SSU extension class reached 3.40 Å after global refinement, improved to 2.88 Å after global and local CTF refinement and 2.71 Å after reference-based motion correction plus global and local CTF refinement.

At this stage, particles from the SSU extension classes of both datasets were merged, resulting in 225,199 particles that refined to 2.52-Å resolution. Focused refinements were then performed using soft masks on the LSU (2.45 Å), followed by local refinements of the LSU L7/12 stalk (3.99 Å) and central protuberance (2.55 Å). Analogous focused refinements were carried out for the SSU (2.63 Å), including separate refinements of the SSU head (2.63 Å), SSU body (2.57 Å) and the SSU extension base (2.98 Å) and tip (3.94 Å).

From these, 175,058 particles had a clear pY factor and 49,141 a P-site tRNA. The P-site tRNA class was refined to 2.84-Å resolution and was further focused refined on the LSU (2.76 Å) and the SSU (2.88 Å). The pY factor class was refined to 2.56-Å resolution and was further focused refined on the LSU (2.48 Å) and the SSU (2.63 Å).

#### Model building and refinement

Focused refined cryo-EM maps (all at resolution better than 3 Å) for the LSU, body and head of the SSU were used as inputs for ModelAngelo^55^ de novo sequencing from the experimental data (without input sequence). Peptide chains obtained were then blasted against *C. reinhardtii* UNIPROT database (taxon ID 3055), and the corresponding AlphaFold2^56^ models were retrieved for all the identified proteins (all were identified). AlphaFold2 models were then matched to the ModelAngelo models using the Matchmaker tool in ChimeraX^57^ and then further rigid-body-fitted in their respective cryo-EM maps. The protein models were then manually inspected and refined in COOT^58^. For the rRNAs, the models from *E. coli* 8B0X^59^ were rigid-body-fitted in the density and then refine with restraints in COOT. The sequence was then mutated accordingly and refined again. For ions, ligands and rRNA modification, they were first placed by homology with 8B0X and then manually curated in COOT. The different parts of the model were then automatically refined in PHENIX against the best resolved focus-refined maps using the phenix.real_space_refine tool and then again manually refined in COOT, this repeating through several cycles. The model geometry was validated using MolProbity^60^ and is summarized in Supplementary Table 1.

### Figure preparation

All figures were prepared using UCSF ChimeraX, developed by the Resource for Biocomputing, Visualization and Informatics at the University of California, San Francisco, with support from National Institutes of Health R01-GM129325 and the Office of Cyber Infrastructure and Computational Biology, National Institute of Allergy and Infectious Diseases^61,62^. For tomogram views with map-backs particle map back, the ArtiaX plugging was used^63^, and membrane segmentations for visualization were automatically performed using MemBrain-seg^64^.

### Reporting summary

Further information on research design is available in the Nature Portfolio Reporting Summary linked to this article.

## Supplementary information

Supplementary Information Contains Supplementary Figs. 1–12 and Tables 1–3.

Reporting summary

Peer Review File

Supplementary Video 1 Sequential sections back and forth through a tomographic volume, followed by a reveal and tour of a chloroplast, showcasing its architecture and chloroplast ribosome identified in this study.

## Data Availability

The single-particle cryo-EM maps of *C. reinhardtii* chlororibosome have been deposited at the Electron Microscopy Data Bank (EMDB) and models on the Protein Data Bank (PDB). For the overall single-particle reconstruction, composite map EMD-56352 (PDB: 9TVU), consensus map EMD-56337, LSU focus EMD-56338, LSU CP focus EMD-56339, LSU L7/12 focus EMD-56340, SSU body EMD-56341 and SSU head EMD-56342, SSU extension base EMD-56349 and SSU extension tip EMD-56344. For the SPA in presence of the factor pY composite map EMD-56602 (PDB: 28LU), consensus map EMD-56560, LSU focus EMD-56563 and SSU focus EMD-56564. For the SPA with the P-site tRNA composite map EMD-56567 (PDB: 28JW), consensus map EMD-56561, LSU focus EMD-56562 and SSU focus EMD-56565. For the subtomogram average of the native *Chlamydomonas* chlororibosome: composite map EMD-56143, consensus map EMD-56139, LSU focus EMD-56140, SSU focus EMD-56141 and SSU extension EMD-56142 and membrane-bound class EMD-56144. The raw cryo-ET data are available at the Electron Microscopy Public Image Archive (EMPIAR), accession code EMPIAR-11830.

## Code availability

Full scripts and analyses for this paper are available via GitHub at https://github.com/Phaips/ChloroRibo.
