# Supplementary material for: Chloroplast-encoded small subunit extensions reshape the Chlamydomonas chlororibosome
Source: Nat Plants. 2026 Jul 27;12(8):1612–21. doi: 10.1038/s41477-026-02361-1 (PMC13481248; doi:10.1038/s41477-026-02361-1)
Supplement: Supplementary file 1 [file 41477_2026_2361_MOESM1_ESM.pdf]

nature plants

Supplementary information

https://doi.org/10.1038/s41477-026-02361-1

Chloroplast-encoded small subunit extensions reshape the *Chlamydomonas* chlororibosome

In the format provided by the authors and unedited

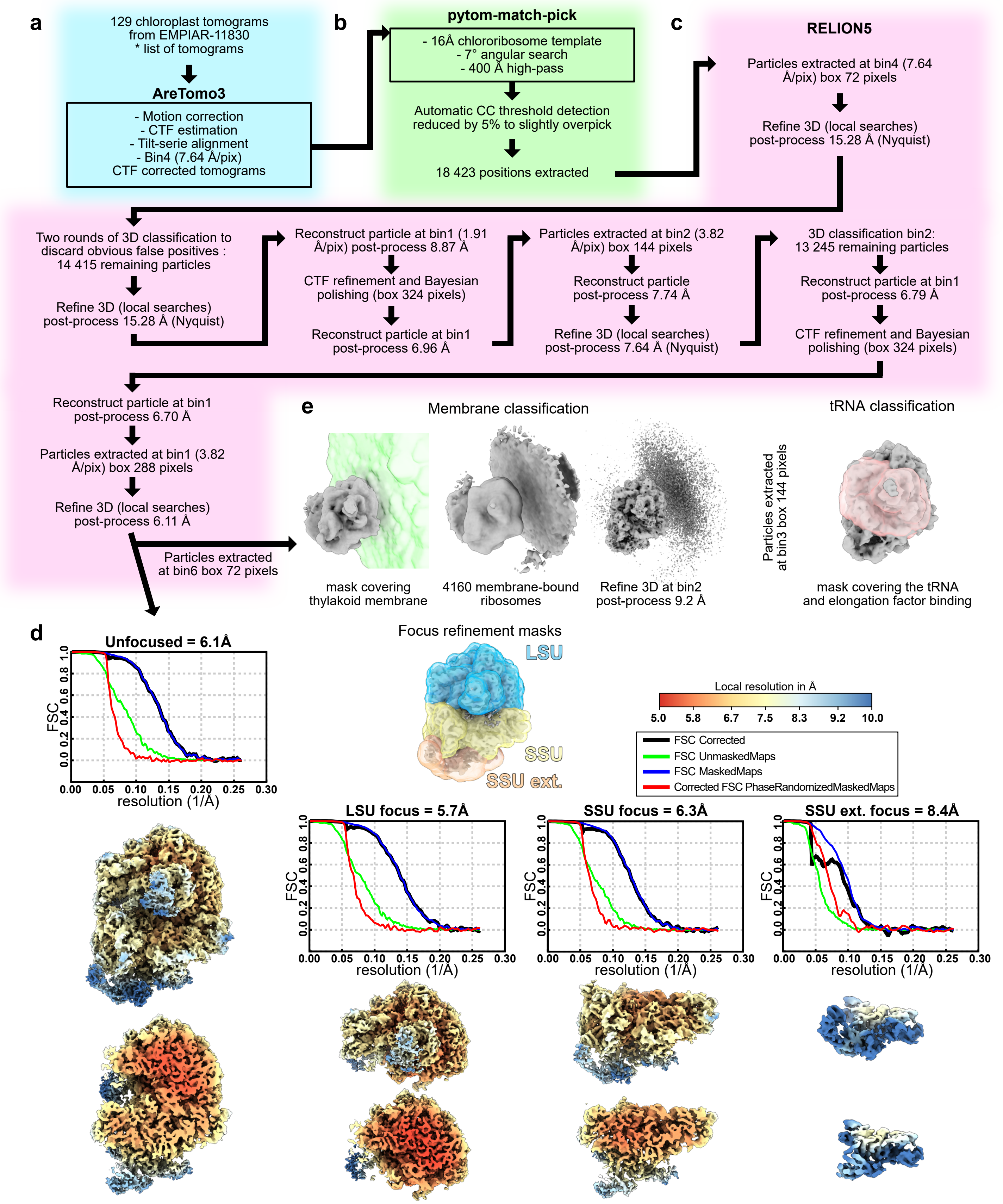

Supplementary Figure 1: Tomography data processing summary

a Tilt-series processing and tomogram reconstruction from 129 chloroplast tomograms (EMPIAR-11830, see Supplementary Table 3) using AreTomo3, including motion correction, CTF estimation, tilt-series alignment, and reconstruction of CTF-corrected tomograms (shown at bin4; 7.64 Å/pixel). **b** Particle picking by 3D template matching in pyTOM-match-pick on bin4 tomograms using a chloroplast ribosome SPA reference low-pass filtered to bin4 Nyquist (15.28 Å), with 6° angular sampling and a 400 Å high-pass. **c** Particle extraction, 3D classification/cleaning, and iterative refinements in RELION5, including re-extraction at higher sampling and CTF refinement/Bayesian polishing. Focused refinements of the LSU, SSU, and SSU-extension were performed using the masks shown. Gold-standard FSC curves are plotted with resolution reported at the FSC = 0.143 criterion. Local-resolution estimates are displayed on the corresponding maps using a common color scale; the local-resolution range for each map is indicated below. Additional 3D classifications used to separate tRNA states and membrane-bound ribosomes (mask-restricted, without alignment) are part of the processing but are not depicted in this schematic.

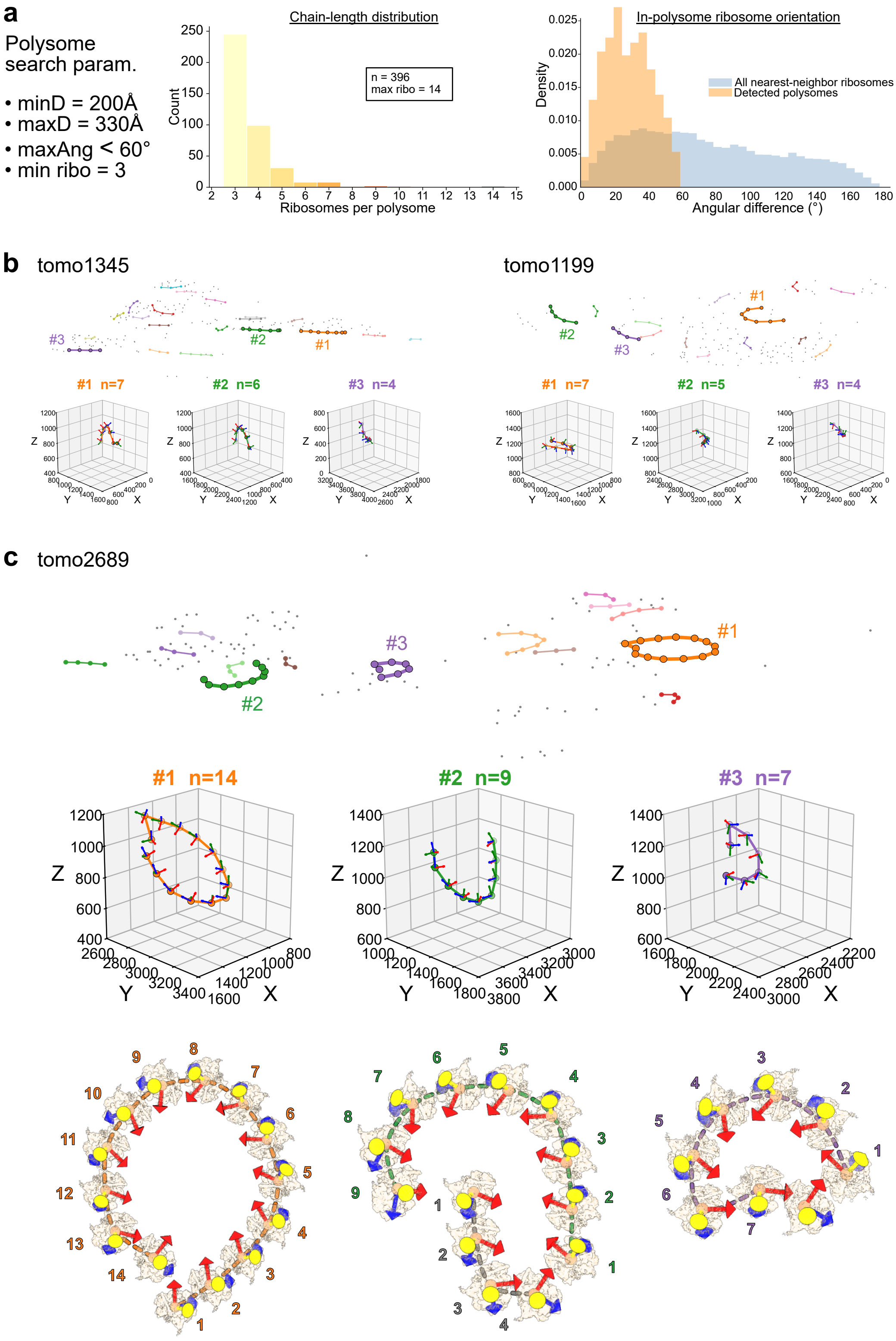

Supplementary Figure 2: Polysome analysis of *in situ* chlororibosomes

a Detection parameters and statistics. Parameters included min/max distances (minD=200Å, maxD=330Å) and <60° angular similarity. With a 3-ribosome minimum, 396 polysomes (max length 14) were detected across 129 tomograms; 151 were found with a 4-ribosome minimum. Left histogram shows chain-length distribution; right plot shows angular differences for all ribosomes (blue) versus polysomal ones (orange). **b** Spatial organization of polysomes in two representative tomograms (1345 and 1199). Top panels show 2D map-backs generated by projecting ribosome coordinates onto their first two principal components. Detected polysomes are color-coded, with the three longest chains numbered and highlighted. Bottom panels show 3D representations of these highlighted polysomes. Individual ribosome orientations are indicated by arrows. Axis dimensions are in pixels. **c** Detailed analysis of polysomes in tomogram 2689. Top and middle panels are the same as described in **b**. Bottom panels show an enlarged map-back of these polysomes using ArtiaX in ChimeraX. Chlororibosome densities are shown with transparency, particle orientations are indicated by arrows, and inferred mRNA paths are represented by dashed lines. The complete methods and output for all tomograms can be found at: https://github.com/Phaips/ChloroRibo.

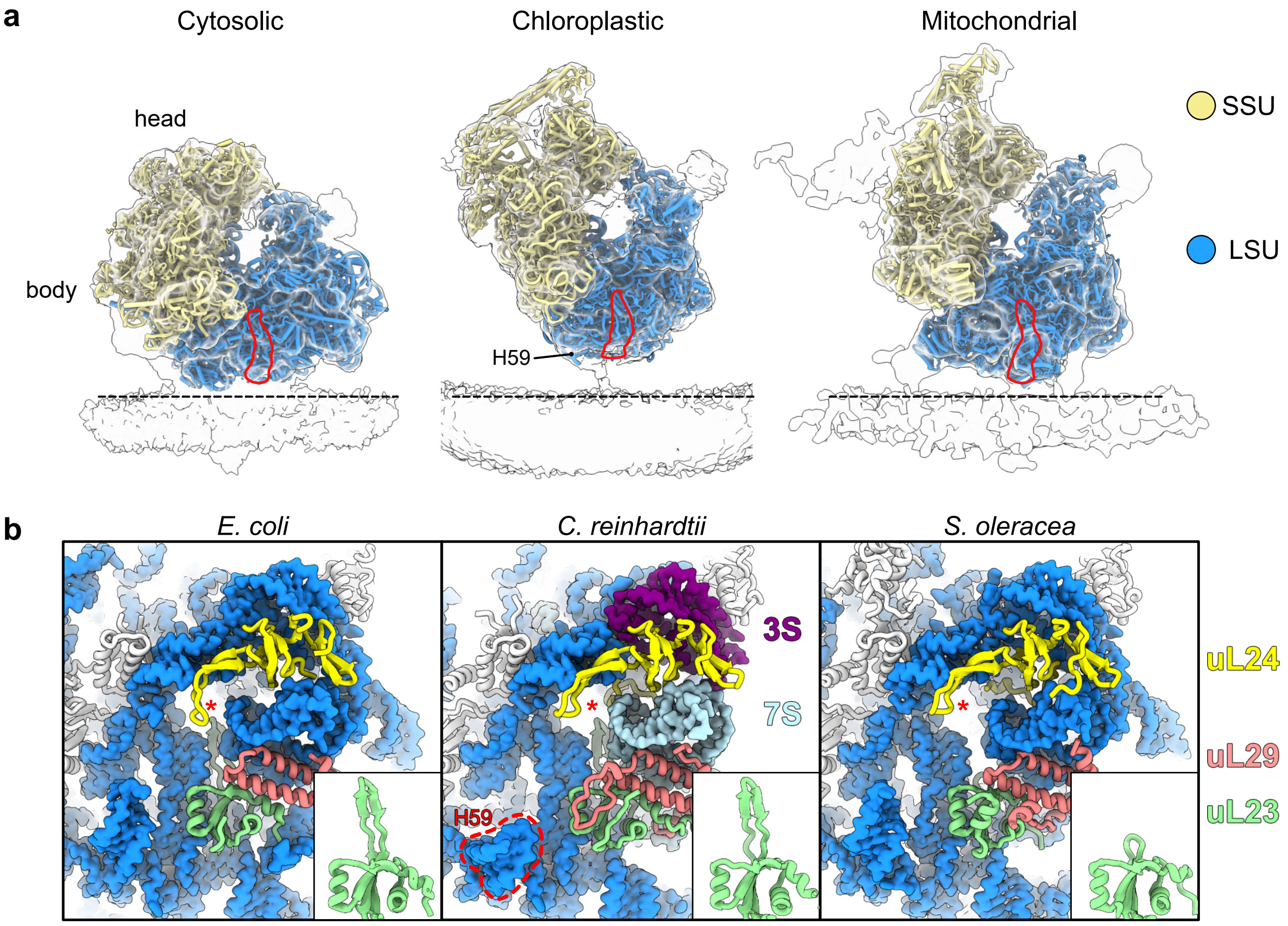

Supplementary Figure 3: Comparative analysis of the membrane association

a Comparison of the membrane association between the cytosolic, chloroplastic and mitochondrial ribosomes in Chlamydomonas. The peptide channel path is highlighted in red. **b** Comparison of the Chlamydomonas chlororibosome area surrounding the peptide exit channel with the E. coli ribosome (PDB: 8B0X) and Spinach chlororibosome (PDB: 6ERI). rRNAs are shown in surface representation and r-proteins in cartoon. The insert shows uL23, notably the shortened loop in the Spinach chlororibosome, that is not occurring in Chlamydomonas.

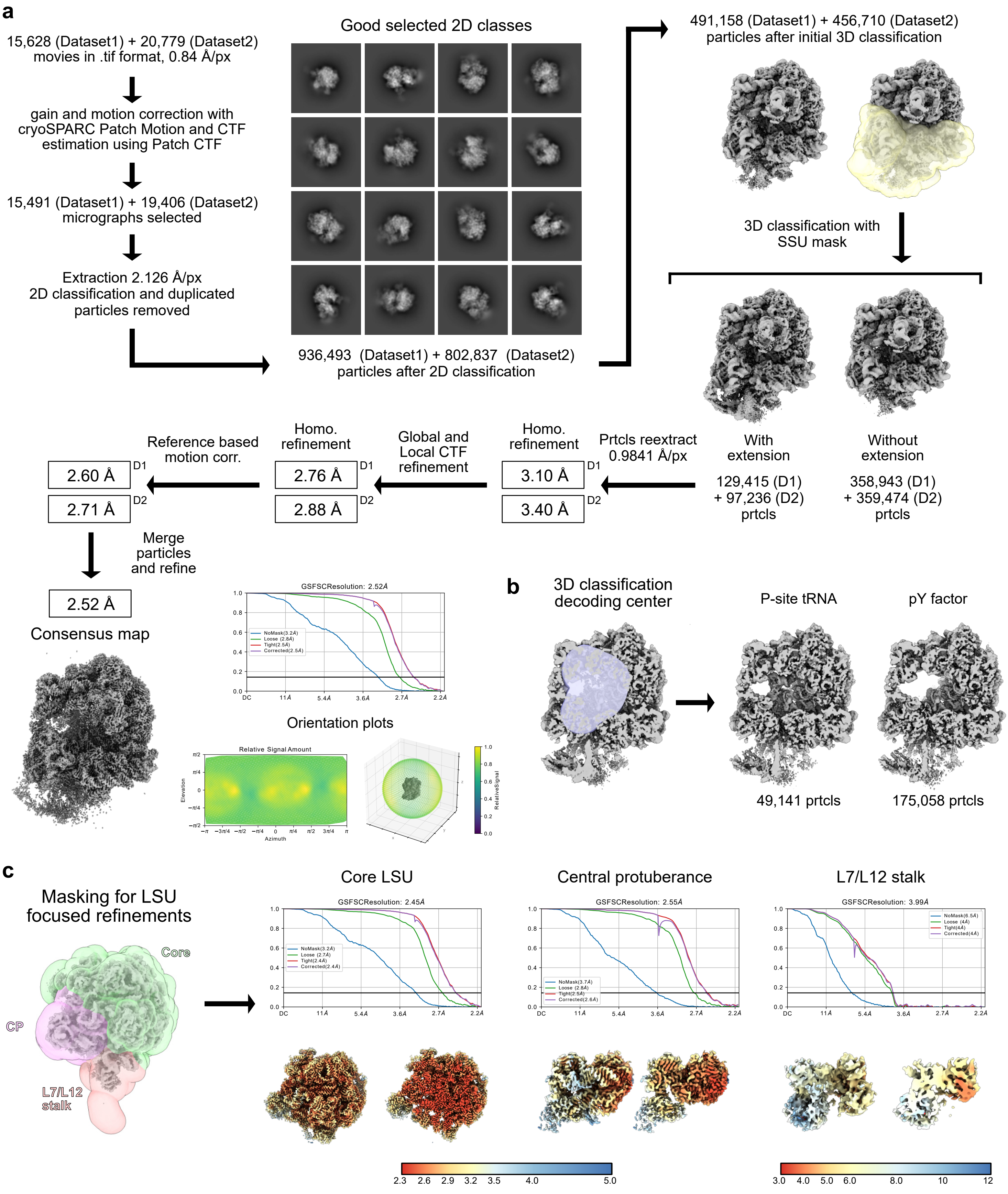

Supplementary Figure 4: Single-particle data processing workflow

Graphical summary of the processing workflow described in Methods. **a** Post-processing, 2D and 3D classification. An orientation plot generated from the Orientation Diagnostic cryoSPARC tool is shown. **b** 3D classification for the decoding area. **c** Focused refinements for the LSU, with all the masks used shown. For the final reconstruction, GSFSC curves are plotted, with resolution calculated at the 0.143 threshold. Local resolution plotted on the maps were generated using the built-in cryoSPARC tool with default parameters, all using the same resolution scale, with maps also shown in cut view.

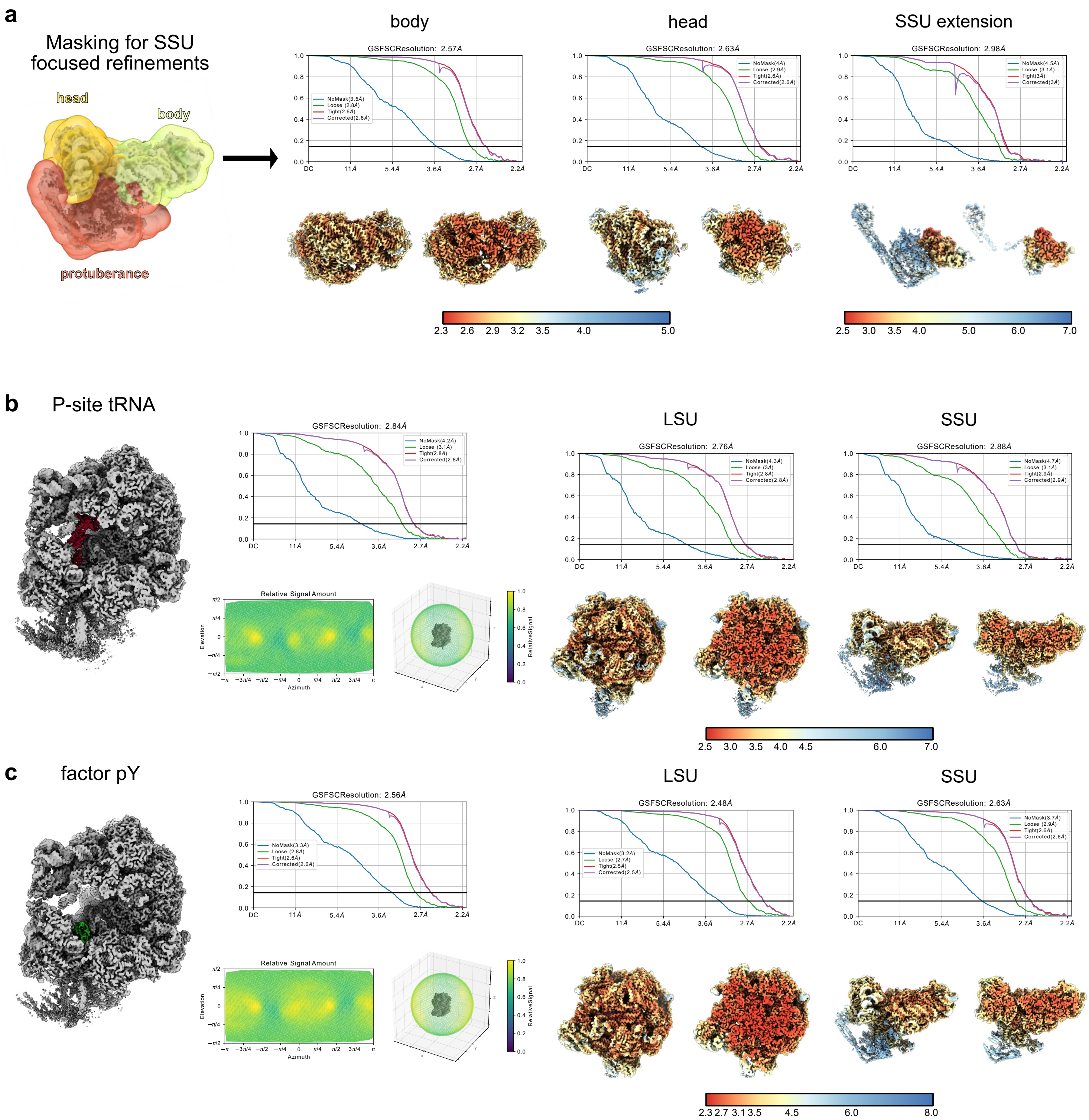

Supplementary Figure 5: Single-particle data processing workflow, FSC plots of the SSU and tRNA and pY states

a Focused refinements for the SSU of the consensus map, with all the masks used shown. For the final reconstruction, GSFSC curves are plotted, with resolution calculated at the 0.143 threshold. Local resolution plotted on the maps were generated using the built-in cryoSPARC tool with default parameters, with a resolution scale different for the SSU head extension, with maps also shown in cut view. **b** and **c** show the consensus refinements for the P-site tRNA and pY factor classes with cryoSPARC orientation plots shown, as well as the respective focused refinements done on the LSU and SSU.

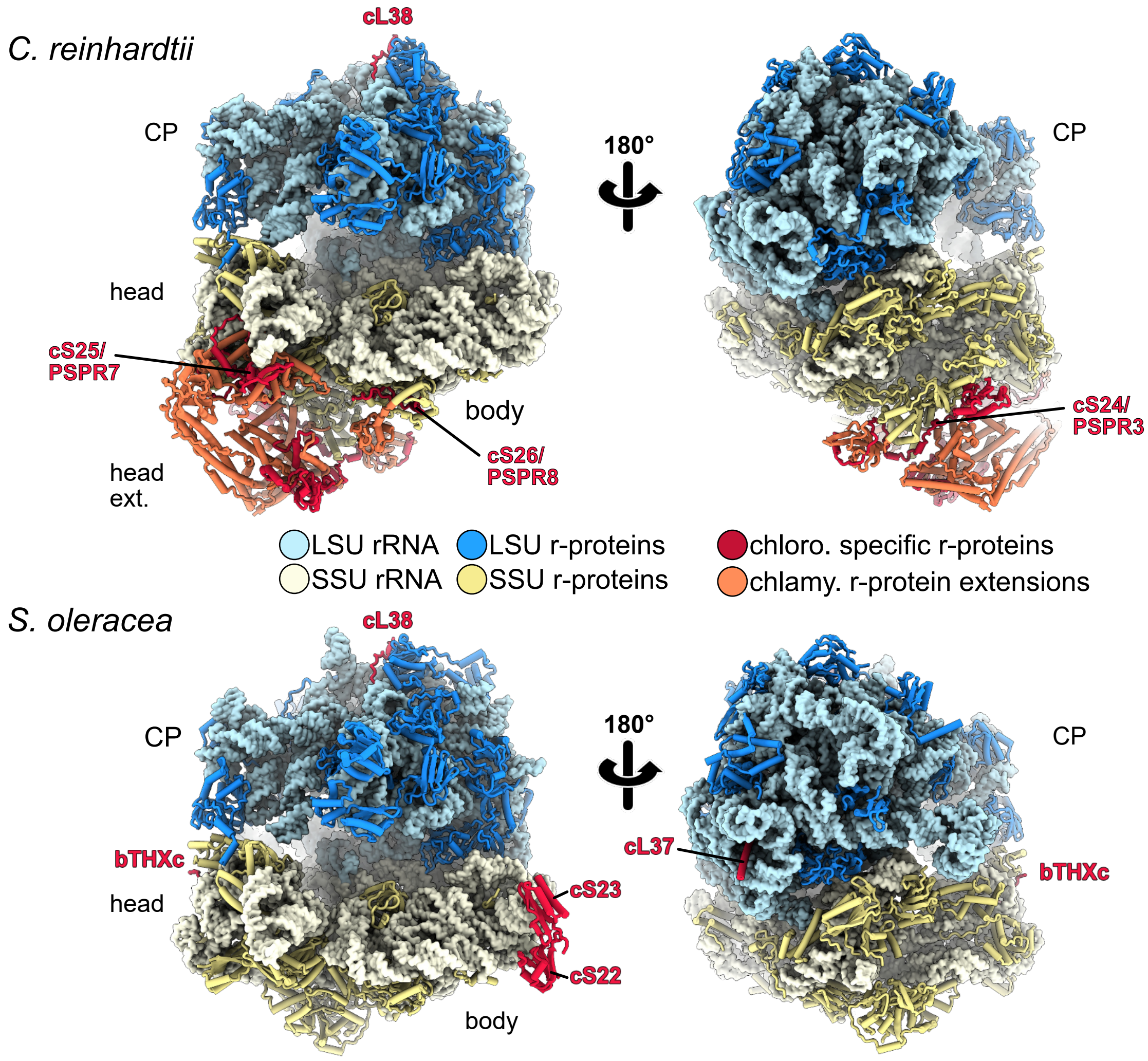

Supplementary Figure 6: Comparison specific r-proteins between Chlamydomonas and Spinach

Chlororibosomes from *C. reinhardtii* (top) and *S. oleracea* (bottom; PDB: 5MMM) are shown in two views related by a 180° rotation. rRNA are shown in surface representation and r-proteins in cartoon and color key is indicated on the figure. Chloroplast-specific r-proteins are highlighted in red and *Chlamydomonas*-specific r-protein extensions in coral. Features of the ribosome are labeled, with CP for central protuberance; “head”, “body”, and “head ext.”.

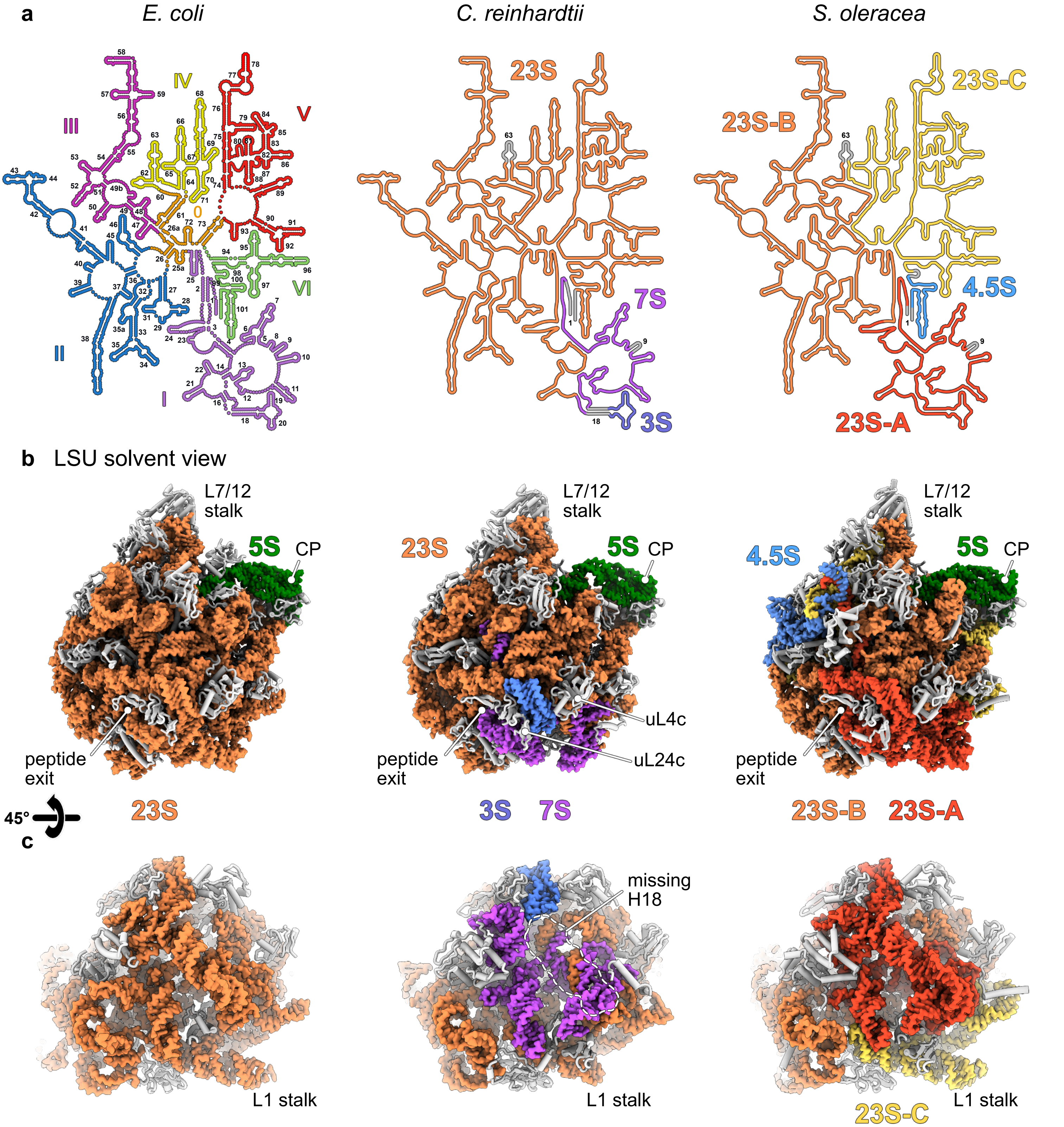

Supplementary Figure 7: Fragmentation of the LSU's rRNAs

a 2D diagrams of the LSU rRNAs from *E. coli*, *C. reinhardtii* and *S. oleracea*. The E. coli one is colored by domains with all helices annotated, while the *C. reinhardtii* and *S. oleracea* ones are colored according to the rRNA fragments with missing helices annotated and grayed out. **b** and **c** shows two different view of the LSU in 3D with rRNAs colored according to **a** and r-proteins shown in white.

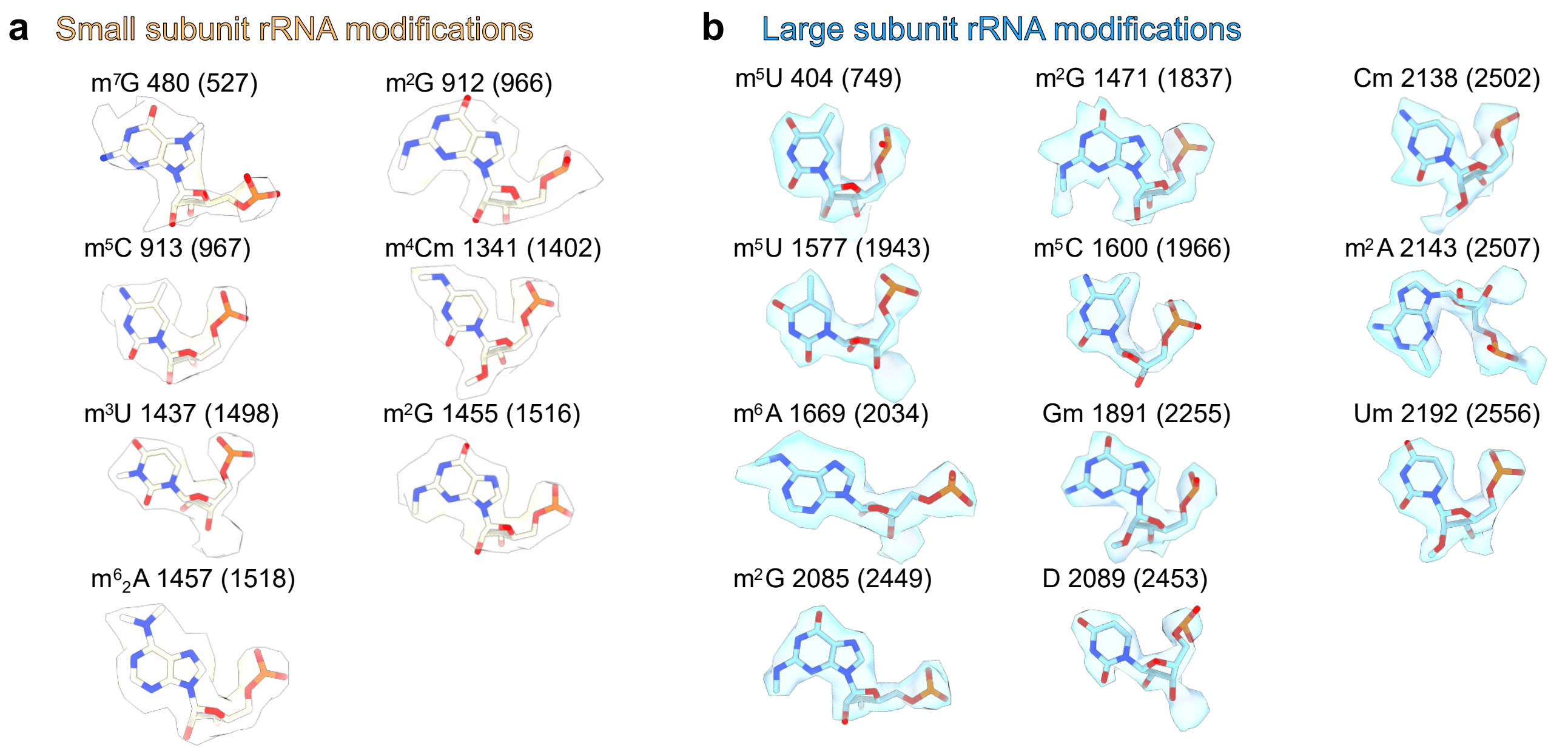

Supplementary Figure 8: Chlamydomonas chlororibosome rRNA modifications

All the rRNA modifications identified from the cryo-EM map are shown in their respective densities with modifications in **a** the LSU 26S rRNA shown in light blue and in **b** the SSU 18S rRNA shown in beige. Numbering in *E. coli* (according to PDB: 8B0X) is shown.

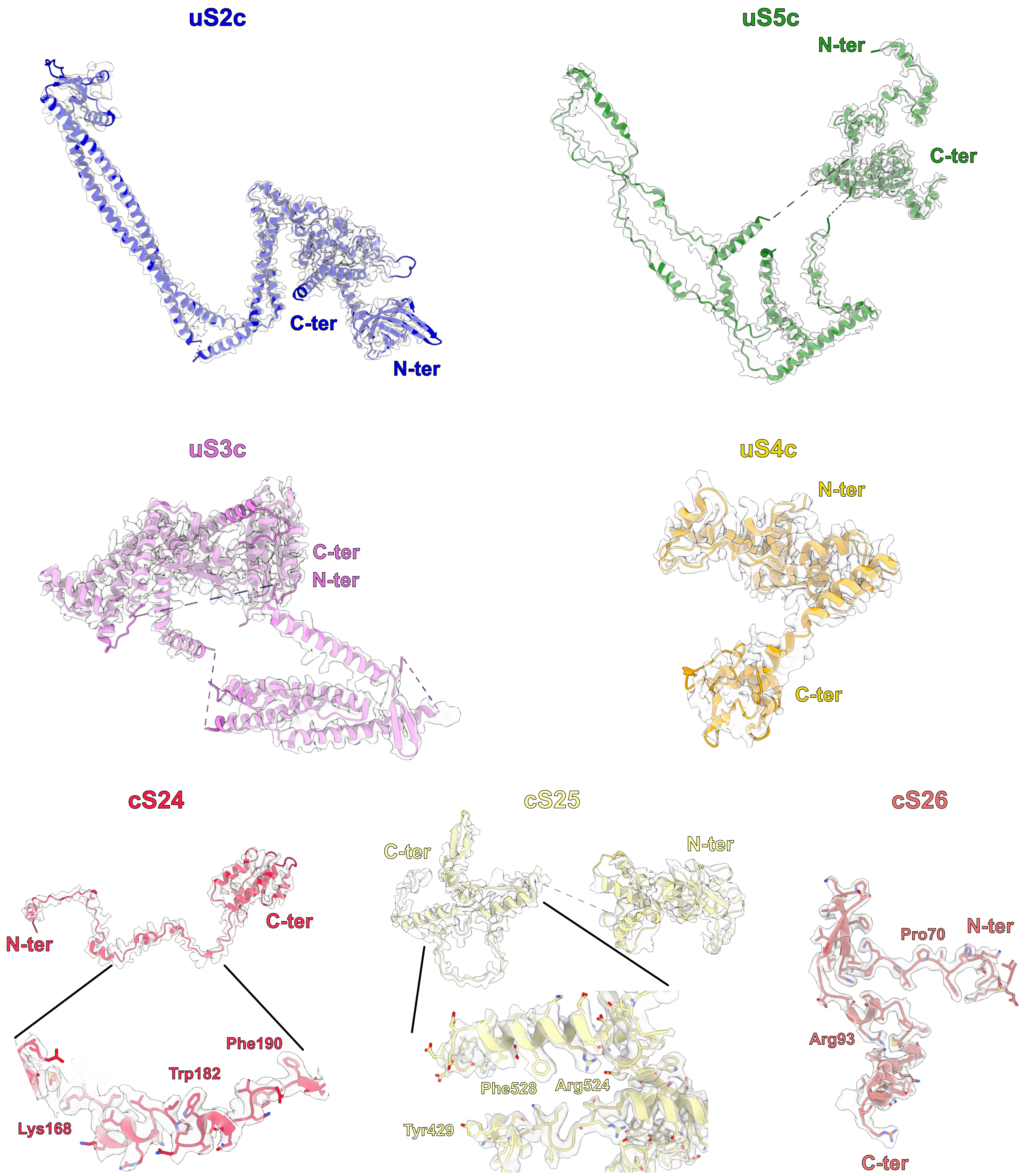

Supplementary Figure 9: Fit of individual arm r-proteins into their densities

Atomic models of the arm constituents are shown as cartoons fitted into their corresponding cryo-EM density maps. The N- and C-termini for each protein are labeled where visible. For cS24, cS25, and cS26, enlarged inset views are provided to demonstrate unambiguous identification of the proteins, with amino acid side chains annotated for reference.

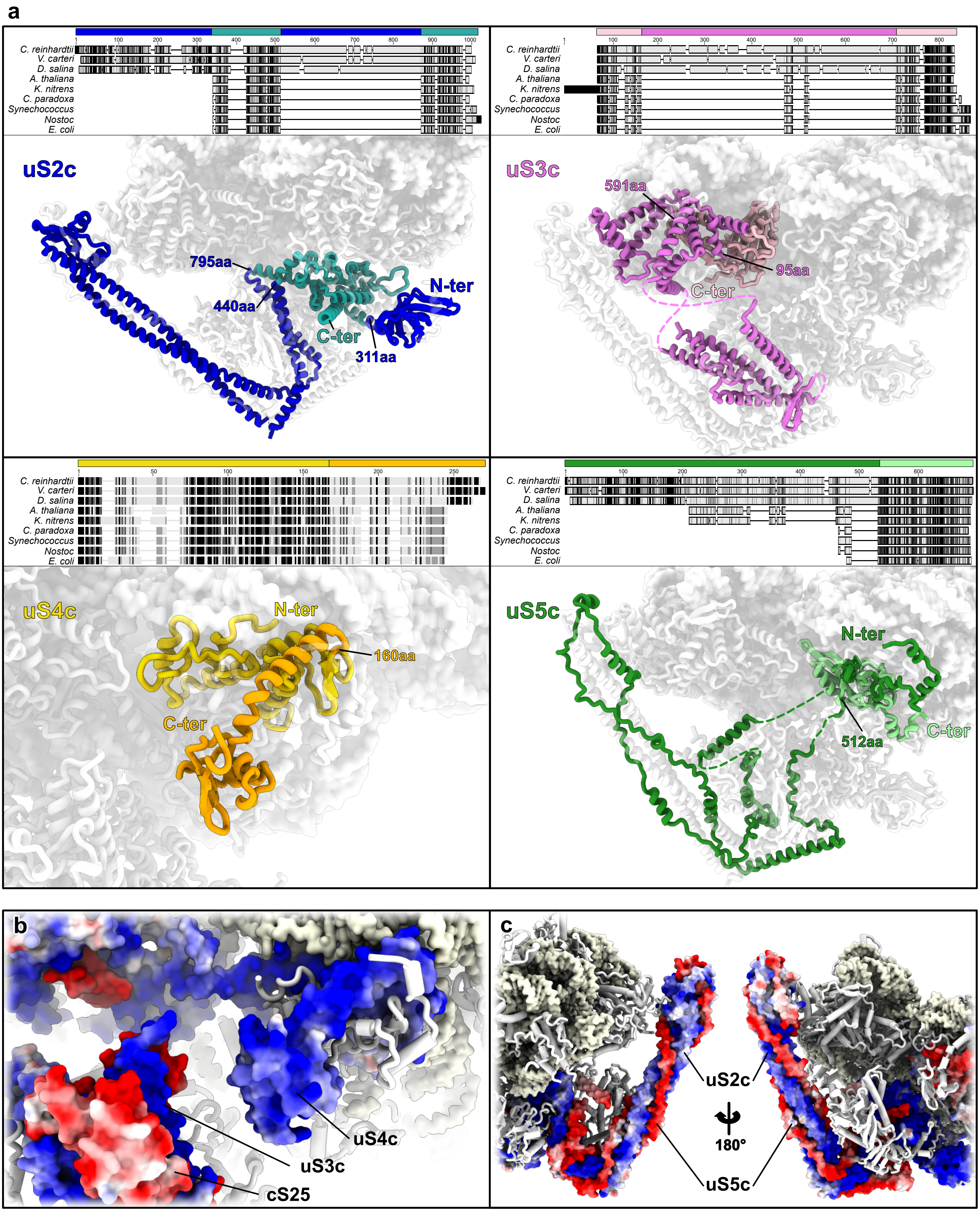

Supplementary Figure 10: Sequence and structural comparison of uS2c, uS3c, uS4c, and uS5c across representative taxa and charge interactions

a Multiple sequence alignments of uS2, uS3, uS4 and uS5 are shown for *Chlamydomonas reinhardtii*, *Volvox carteri*, and *Dunaliella salina* (Chlorophyceae); *Arabidopsis thaliana* and *Klebsormidium nitens* (Streptophyta); *Cyanophora paradoxa* (Glaucophyta); *Synechococcus elongatus* and *Nostoc sp.* PCC 7107 (Cyanobacteria); and *Escherichia coli*. Conserved core regions of each ribosomal protein are depicted in lighter shades, while algal-specific insertions and extensions are shown in darker colors, both on the 3D structures and the alignments. Unmodeled parts of the proteins are shown as dashed line. The boundaries of these extensions are annotated on the structures. uS2c (910aa) presents a 356aa insertion and a 310aa N-ter extension, uS3c (712aa) includes a 497aa insertion, uS4c (257aa) features a 97aa C-ter extension and uS5c (673aa) displays a 512aa N-ter extension. **b, c** proteins uS3c, uS4c and cS25 (**b**) as well as uS2c and uS5c (**c**) represented as surface and colored by electrostatic potential, with blue positively charged and red negatively charged.

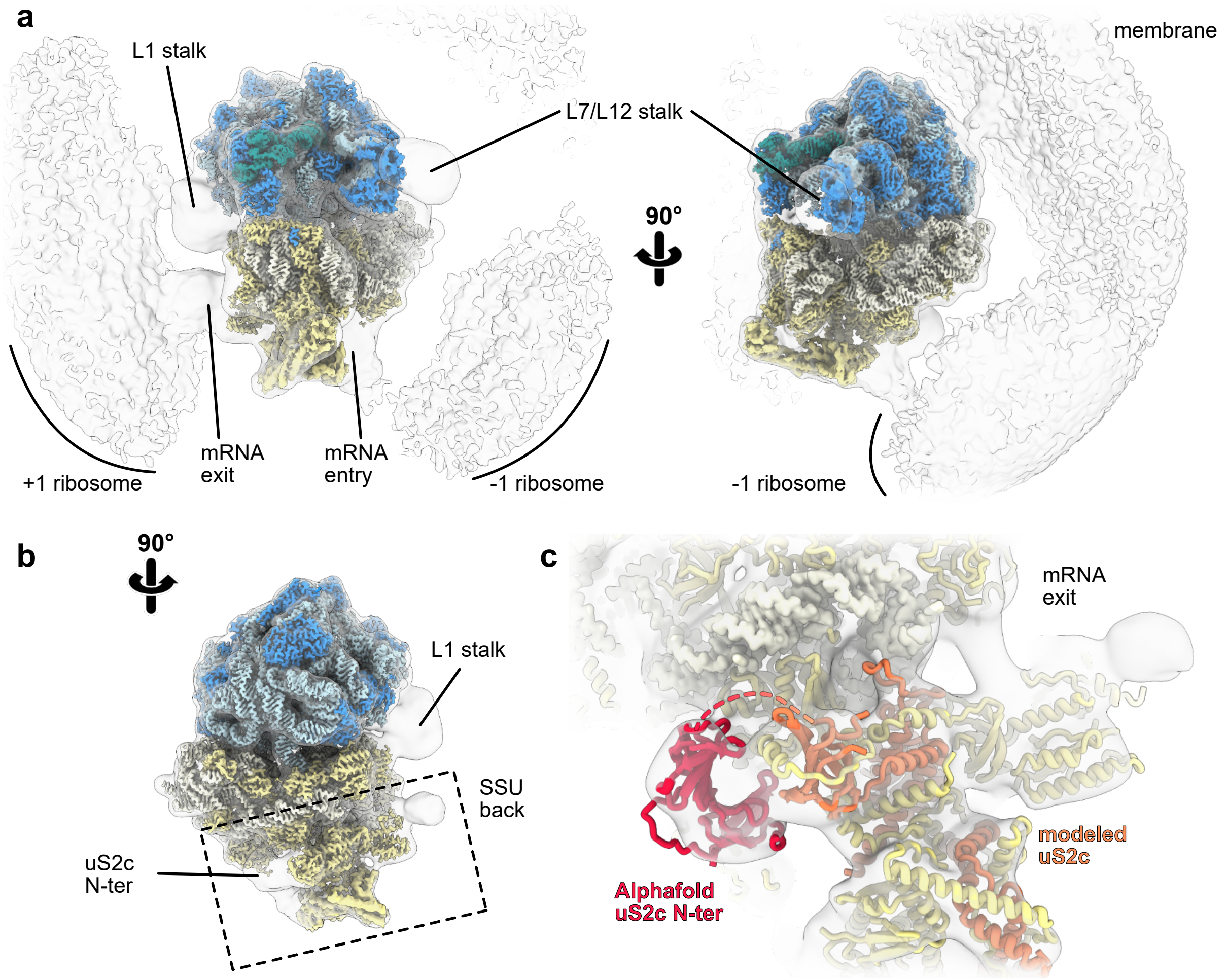

Supplementary Figure 11: Comparison between SPA and STA reconstructions of the chlororibosome

a The SPA map (colored by component: LSU rRNA, light blue; LSU ribosomal proteins, dark blue; SSU rRNA, light yellow; SSU ribosomal proteins, yellow) is shown alongside a low-pass–filtered consensus STA map (grey). Two orientations are shown, highlighting features present in STA but absent or attenuated in SPA, including additional +1 and −1 chlororibosomes within polysomes, membrane-associated density, and the L1 and L7/L12 stalk regions. **b** View of the SSU back (boxed, dashed outline), rotated 90° relative to a, revealing an unoccupied density at the expected position of the uS2c N-terminus. **c** Close-up of the SSU-back region near the mRNA exit site. The fitted chlororibosome model is shown in yellow, with the modeled uS2c segment in coral, and an AlphaFold prediction for the uS2c N-ter (residues 23–168) shown in crimson, comprising two β-barrel domains.

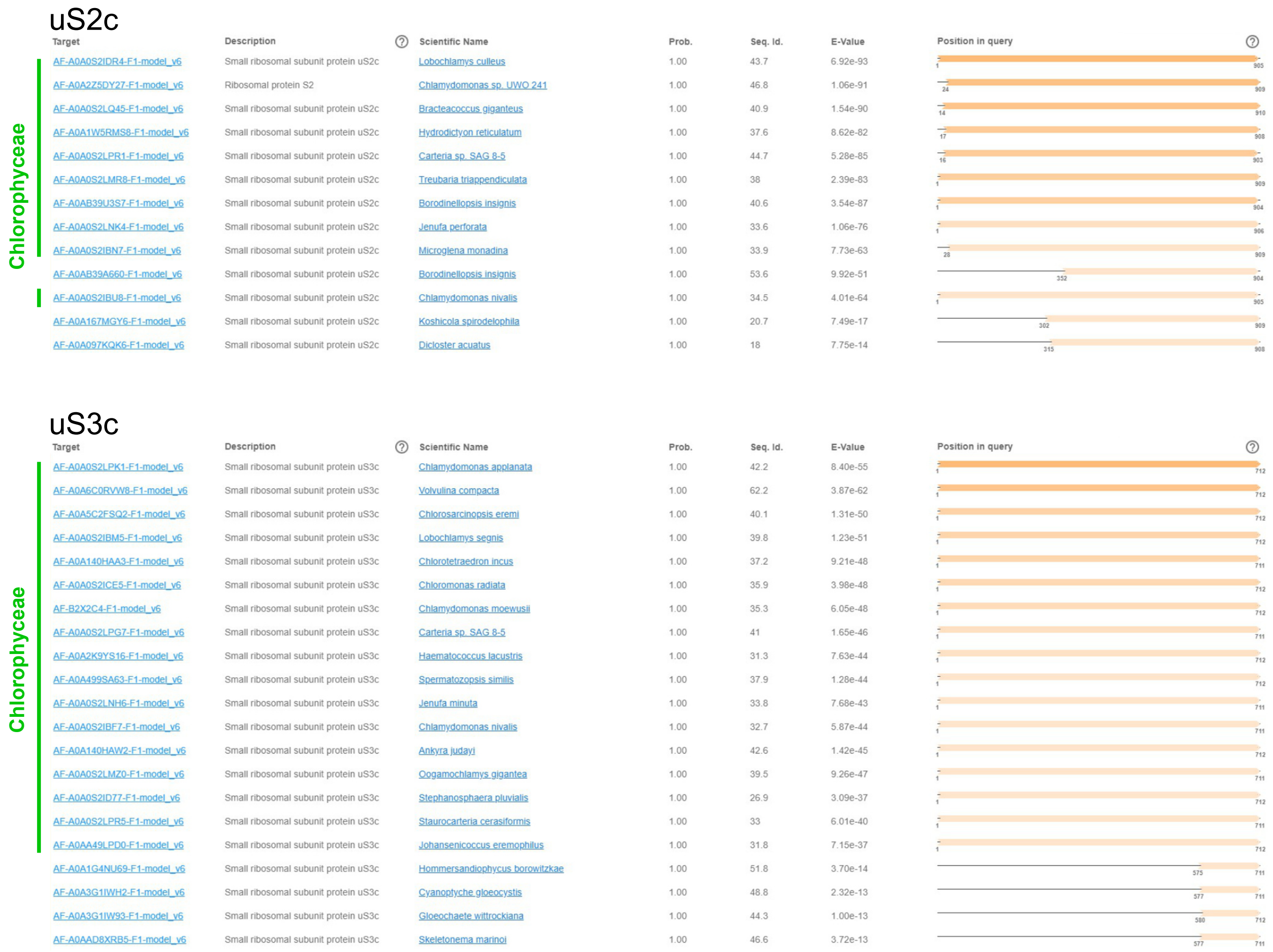

uS2c

Target	Description	Scientific Name	Prob.	Seq. Id.	E-Value	Position in query
AF-A0A0S2IDR4-F1-model_v6	Small ribosomal subunit protein uS2c	Lobochlamys culleus	1.00	43.7	6.92e-93	1–905
AF-A0A2Z5DY27-F1-model_v6	Ribosomal protein S2	Chlamydomonas sp. UWO 241	1.00	46.8	1.06e-91	24–909
AF-A0A0S2LQ45-F1-model_v6	Small ribosomal subunit protein uS2c	Bracteacoccus giganteus	1.00	40.9	1.54e-90	14–910
AF-A0A1W5RMS8-F1-model_v6	Small ribosomal subunit protein uS2c	Hydrodictyon reticulatum	1.00	37.6	8.62e-82	17–908
AF-A0A0S2LPR1-F1-model_v6	Small ribosomal subunit protein uS2c	Carteria sp. SAG 8-5	1.00	44.7	5.28e-85	16–903
AF-A0A0S2LMR8-F1-model_v6	Small ribosomal subunit protein uS2c	Treubaria triappendiculata	1.00	38	2.39e-83	1–909
AF-A0AB39U3S7-F1-model_v6	Small ribosomal subunit protein uS2c	Borodinellopsis insignis	1.00	40.6	3.54e-87	1–904
AF-A0A0S2LNK4-F1-model_v6	Small ribosomal subunit protein uS2c	Jenufa perforata	1.00	33.6	1.06e-76	1–906
AF-A0A0S2IBN7-F1-model_v6	Small ribosomal subunit protein uS2c	Microglena monadina	1.00	33.9	7.73e-63	28–909
AF-A0AB39A660-F1-model_v6	Small ribosomal subunit protein uS2c	Borodinellopsis insignis	1.00	53.6	9.92e-51	352–904
AF-A0A0S2IBU8-F1-model_v6	Small ribosomal subunit protein uS2c	Chlamydomonas nivalis	1.00	34.5	4.01e-64	1–905
AF-A0A167MGY6-F1-model_v6	Small ribosomal subunit protein uS2c	Koshicola spirodelophila	1.00	20.7	7.49e-17	302–909
AF-A0A097KQK6-F1-model_v6	Small ribosomal subunit protein uS2c	Dicloster acuatus	1.00	18	7.75e-14	315–908

uS3c

Target	Description	Scientific Name	Prob.	Seq. Id.	E-Value	Position in query
AF-A0A0S2LPK1-F1-model_v6	Small ribosomal subunit protein uS3c	Chlamydomonas applanata	1.00	42.2	8.40e-55	1–712
AF-A0A6C0RVW8-F1-model_v6	Small ribosomal subunit protein uS3c	Volvulina compacta	1.00	62.2	3.87e-62	1–712
AF-A0A5C2FSQ2-F1-model_v6	Small ribosomal subunit protein uS3c	Chlorosarcinopsis eremi	1.00	40.1	1.31e-50	1–712
AF-A0A0S2IBM5-F1-model_v6	Small ribosomal subunit protein uS3c	Lobochlamys segnis	1.00	39.8	1.23e-51	1–712
AF-A0A140HAA3-F1-model_v6	Small ribosomal subunit protein uS3c	Chlorotetraedron incus	1.00	37.2	9.21e-48	1–711
AF-A0A0S2ICE5-F1-model_v6	Small ribosomal subunit protein uS3c	Chloromonas radiata	1.00	35.9	3.98e-48	1–712
AF-B2X2C4-F1-model_v6	Small ribosomal subunit protein uS3c	Chlamydomonas moewusii	1.00	35.3	6.05e-48	1–712
AF-A0A0S2LPG7-F1-model_v6	Small ribosomal subunit protein uS3c	Carteria sp. SAG 8-5	1.00	41	1.65e-46	1–711
AF-A0A2K9YS16-F1-model_v6	Small ribosomal subunit protein uS3c	Haematococcus lacustris	1.00	31.3	7.63e-44	1–712
AF-A0A499SA63-F1-model_v6	Small ribosomal subunit protein uS3c	Spermatozopsis similis	1.00	37.9	1.28e-44	1–712
AF-A0A0S2LNH6-F1-model_v6	Small ribosomal subunit protein uS3c	Jenufa minuta	1.00	33.8	7.68e-43	1–711
AF-A0A0S2IBF7-F1-model_v6	Small ribosomal subunit protein uS3c	Chlamydomonas nivalis	1.00	32.7	5.87e-44	1–711
AF-A0A140HAW2-F1-model_v6	Small ribosomal subunit protein uS3c	Ankyra judayi	1.00	42.6	1.42e-45	1–712
AF-A0A0S2LMZ0-F1-model_v6	Small ribosomal subunit protein uS3c	Oogamochlamys gigantea	1.00	39.5	9.26e-47	1–711
AF-A0A0S2ID77-F1-model_v6	Small ribosomal subunit protein uS3c	Stephanosphaera pluvialis	1.00	26.9	3.09e-37	1–712
AF-A0A0S2LPR5-F1-model_v6	Small ribosomal subunit protein uS3c	Staurocarteria cerasiformis	1.00	33	6.01e-40	1–711
AF-A0AA49LPD0-F1-model_v6	Small ribosomal subunit protein uS3c	Johansenicoccus eremophilus	1.00	31.8	7.15e-37	1–712
AF-A0A1G4NU69-F1-model_v6	Small ribosomal subunit protein uS3c	Hommersandiophycus borowitzkae	1.00	51.8	3.70e-14	575–711
AF-A0A3G1IWH2-F1-model_v6	Small ribosomal subunit protein uS3c	Cyanoptyche gloeocystis	1.00	48.8	2.32e-13	577–711
AF-A0A3G1IW93-F1-model_v6	Small ribosomal subunit protein uS3c	Gloeochaete wittrockiana	1.00	44.3	1.00e-13	580–712
AF-A0AAD8XRB5-F1-model_v6	Small ribosomal subunit protein uS3c	Skeletonema marinoi	1.00	46.6	3.72e-13	577–711

Supplementary Figure 12: Conservation of extra SSU domain (FoldSeek)

Foldseek was used to query the predicted structures of *C. reinhardtii* uS2c (top) and uS3c (bottom). Only uS2 and uS3 are shown because they presented clearly folded regions as opposed to the extensions of the other proteins that are more disordered. For each query, the table reports the top-ranked hits with the corresponding organism, Foldseek probability score, sequence identity, and E-value. This illustrates the conservation of uS2c and uS3c in chlorophyceae.

	Full high res. (PDB 9TVU - EMD-56352)	P-site tRNA (PDB 28JW - EMD-56567)	pY factor (PDB 28LU - EMD-56602)
	Unfocused	Unfocused	Unfocused
EMDB	EMD-56337	EMD-56561	EMD-56560
**Data collection and processing**			
Magnification	75,000x	75,000x	75,000x
Voltage (kV)	300	300	300
Electron exposure (e–/Å^2)	50	50	50
Defocus range (μm)	-0.8 to -2.0	-0.8 to -2.0	-0.8 to -2.0
Pixel size (Å)	0.84	0.84	0.84
Symmetry imposed	C1	C1	C1
Initial particle images (no.)	1,739,330	1,739,330	1,739,330
Final particle images (no.)	225,199	49,141	175,058
Map resolution (Å) - FSC 0.143	2.52	2.84	2.56
Map resolution range (Å)	2.3 - 5.8	2.4 - 7.5	2.3 - 6.2
**Refinement**			
Initial model used (PDB code)	AF2	AF2	AF2
FSC model (0.143)	2.5	2.8	2.6
CC Model vs. Data (mask)	0.83	0.82	0.82
Map sharpening *B* factor (Å^2)	-54.7	-32.9	-51.9
Model composition			
Non-hydrogen atoms	158411	160218	159564
Residues: Protein - Nucleotide	8399 - 4285	8406 - 4367	8545 - 4285
Water	-	-	-
Ligands	K: 138<br>MG: 323	K: 130<br>MG: 319<br>CLM:1	K: 138<br>MG: 322
B factors (Å^2)			
Protein	0.25/422.72/60.49	1.47/442.59/85.12	0.00/312.71/68.79
Nucleotide	0.42/231.80/32.59	0.22/240.78/59.65	1.18/185.65/43.56
Ligand	6.73/133.09/31.00	20.56/119.88/52.06	7.9/138.32/38.73
R.m.s. deviations			
Bond lengths (Å)	0.009	0.007	0.007
Bond angles (°)	0.848	0.802	0.771
Validation			
MolProbity score	1.68	1.39	1.64
Clashscore	5.35	4.77	4.41
Poor rotamers (%)	1.77	2.05	2.20
Ramachandran plot			
Favored (%)	96.76	96.76	97.11
Allowed (%)	3.11	3.11	2.78
Disallowed (%)	0.13	0.13	0.11

Supplementary Table 1: Cryo-EM statistics table

Name	UNIPROT ID	Genome	Size	Location	Chain name	Comment
bS1c	Q70DX8	Cre09.g394750	436	back	a	
uS2c	A0A218N8X3	CreCp.g802299	910	back	b	SSU arm extension
uS3c	Q08365	CreCp.g802310	712	head	c	SSU arm extension
uS4c	P48270	CreCp.g802283	257	body	d	SSU arm extension
uS5c	A8J8M5	Cre16.g659950	673	body	e	SSU arm extension
bS6c	A8J5Y7	Cre12.g520600	171	body	f	
uS7c	P48267	CreCp.g802288	168	head	g	
uS8c	P59775	CreCp.g802279	141	body	h	
uS9c	O20029	CreCp.g802303	191	head	i	
uS10c	A8IWQ1	Cre03.g195650	169	head	j	N-ter contacts uS2c and uS5c ext.
uS11c	Q7YKX3	CreCp.g802319	130	body	k	
uS12c	P14149	CreCp.g802325	133	body	l	
uS13c	A8JDP6	Cre12.g493950	164	head	m	
uS14c	Q9GGE2	CreCp.g802289	100	head	n	
uS15c	A8HWZ8	Cre06.g264300	141	body	o	
bS16c	A8JDN8	Cre12.g494450	128	body	p	
uS17c	A8JGS2	Cre02.g118950	105	body	q	
bS18c	O20032	CreCp.g802300	137	body	r	
uS19c	P59776	CreCp.g802275	92	head	s	
bS20c	A8JDN4	Cre12.g494750	166	body	t	
bS21c	A8HPN4	Cre01.g017300	184	body	u	
cS24/PSRP3	A8I8A3	Cre02.g083950	298	back	v	interactions with uS2c and bS1c
cS25/PSRP7	Q5QEB1	Cre12.g519180	560	head	w	interactions with uS3c extensions
cS26/PSRP8	A8I8A6	Cre02.g084000	120	body	x	replaces C-ter of uS4c
pY/PSRP1	A8J8B2	Cre05.g237450	285		y	only in hibernation
uL1c	A8I8Z4	Cre02.g088900	-	L1 stalk	A	not resolved
uL2c	Q8HTL2	CreCp.g802274	278		B	
uL3c	A8JE35	Cre48.g761197	259		C	
uL4c	Q84U22	Cre11.g479500	243		D	
uL5c	Q8HTL1	CreCp.g802278	179	CP	E	
uL6c	A8J503	Cre09.g415950	207		F	
bL9c	A0A2K3D5T7	Cre12.g556050	200		G	
uL10c	A8HVP7	Cre06.g272850	235	L7/L12 stalk	H	
uL11c	A8ICE4	Cre10.g423650	176	L7/L12 stalk	I	
uL13c	A8HWZ6	Cre06.g264350	225		J	
uL14c	P11094	CreCp.g802277	122		K	
uL15c	A8JAL6	Cre14.g612450	241		L	
uL16c	P05726	CreCp.g802276	136		M	
bL17c	A8I3M4	Cre02.g108850	173		N	
uL18c	A8HNJ8	Cre01.g052100	145	CP	O	
bL19c	A8IW44	Cre17.g734450	153		P	
bL20c	P26565	CreCp.g802268	112		Q	
bL21c	A8IP00	Cre06.g299000	179		R	
uL22c	Q84U21	Cre13.g580850 ?	175		S	
uL23c	Q8HTL3	CreCp.g802273	95		T	
uL24c	A8J9D9	Cre16.g652550	170		U	
bL27c	A8INR7	Cre06.g300800	161		V	
bL28c	A8HWS8	Cre06.g265800	195		W	
uL29c	A8HXM1	Cre06.g259850	134		X	
bL31c	A8J3Z3	Cre08.g365400	136	CP	Y	
bL32c	A8IUC3	Cre07.g352850	98		Z	
bL33c	A8I1D3	Cre10.g462950	101		6	
bL34c	A8HQG3	Cre01.g030050	124		7	
bL35c	A8JEP1	Cre04.g217932	114		8	
bL36c	P59774	CreCp.g802272	37		9	
cL38/PSRP6	A8IMN3	Cre06.g308533	66		0	

Supplementary Table 2: Ribosomal proteins of the *Chlamydomonas reinhardtii* chloroplast ribosome

Proteins are grouped by subunit (SSU, yellow; LSU, blue); chloroplast-specific proteins are highlighted in red. For each protein, the UniProt accession, gene model identifier (C.reinhardtii_CC-4532 v6.1 genome, with green indicating chloroplast encoded proteins), protein length (aa), assigned structural location, chain identifier used in the model, and relevant notes are listed.

tomo11	gain1
tomo12	gain1
tomo23	gain1
tomo31	gain1

tomo87	gain2
tomo92	gain2
tomo182	gain2
tomo200	gain2
tomo202	gain2
tomo205	gain2
tomo242	gain2
tomo243	gain2
tomo257	gain2
tomo284	gain2
tomo317	gain2
tomo318	gain2
tomo341	gain2
tomo342	gain2
tomo364	gain2
tomo373	gain2
tomo394	gain2
tomo408	gain2
tomo436	gain2
tomo444	gain2
tomo446	gain2
tomo448	gain2

tomo462	gain3
tomo473	gain3
tomo499	gain3
tomo517	gain3
tomo518	gain3
tomo555	gain3
tomo573	gain3
tomo576	gain3
tomo595	gain3
tomo611	gain3
tomo632	gain3
tomo663	gain3
tomo679	gain3
tomo683	gain3

Gain references:

gain1	20211216_124009_EER_GainReference.gain
gain2	20220427_000718_EER_GainReference.gain
gain3	20220717_000157_EER_GainReference.gain
gain4	20220717_000157_EER_GainReference.gain
gain5	20230807_182744_EER_GainReference.gain
gain6	20230811_112217_EER_GainReference.gain
gain7	20230903_185905_EER_GainReference_NNPKrios.gain

tomo846	gain4
tomo867	gain4
tomo868	gain4
tomo869	gain4
tomo900	gain4
tomo909	gain4
tomo911	gain4
tomo912	gain4
tomo944	gain4
tomo949	gain4
tomo1034	gain4
tomo1119	gain4
tomo1132	gain4
tomo1168	gain4
tomo1182	gain4
tomo1184	gain4
tomo1198	gain4
tomo1199	gain4
tomo1212	gain4
tomo1221	gain4
tomo1272	gain4
tomo1274	gain4
tomo1283	gain4
tomo1285	gain4
tomo1286	gain4
tomo1309	gain4
tomo1310	gain4
tomo1311	gain4
tomo1315	gain4
tomo1332	gain4
tomo1334	gain4
tomo1335	gain4
tomo1341	gain4
tomo1343	gain4
tomo1345	gain4

tomo1402	gain5
tomo1405	gain5
tomo1533	gain5

tomo1711	gain6
tomo1728	gain6
tomo1743	gain6
tomo1836	gain6
tomo1855	gain6
tomo1868	gain6
tomo2325	gain6
tomo2358	gain6
tomo2362	gain6
tomo2449	gain6
tomo2453	gain6
tomo2666	gain6
tomo2668	gain6
tomo2669	gain6
tomo2680	gain6
tomo2689	gain6
tomo2730	gain6
tomo2787	gain6
tomo2824	gain6
tomo2828	gain6

tomo2068	gain7
tomo2122	gain7
tomo2148	gain7
tomo2152	gain7
tomo2154	gain7
tomo2164	gain7
tomo2183	gain7
tomo2190	gain7
tomo2196	gain7
tomo2203	gain7
tomo2204	gain7
tomo2212	gain7
tomo2245	gain7
tomo2271	gain7
tomo2278	gain7
tomo2283	gain7
tomo2285	gain7

tomo2839	gain7_2°
tomo2871	gain7_2°
tomo2879	gain7_2°
tomo2882	gain7_2°
tomo2885	gain7_2°
tomo2893	gain7_2°
tomo2895	gain7_2°
tomo2902	gain7_2°
tomo2904	gain7_2°
tomo2905	gain7_2°
tomo2908	gain7_2°
tomo2914	gain7_2°
tomo2942	gain7_2°
tomo2946	gain7_2°

Supplementary Table 3: List of tomograms used from EMPIAR-11830

List of the tomograms used from the publicly deposited EMPIAR-11830. The tomograms are broken down by gain reference, and the last ones were collected with a 2° tilt increment, contrary to the rest that were collected with a 3° increment. The correspondance between the tomogram numbers and their raw names in the deposited can be found at https://github.com/Chromatin-Structure-Rhythms-Lab/ChlamyAnnotations.
