# Supplementary material for: Chloroplast-encoded small subunit extensions reshape the Chlamydomonas chlororibosome
Source: Nat Plants. 2026 Jul 27;12(8):1612–21. doi: 10.1038/s41477-026-02361-1 (PMC13481248; doi:10.1038/s41477-026-02361-1)
Supplement: Supplementary file 2 [file 41477_2026_2361_MOESM2_ESM.pdf]

nature portfolio

Corresponding author(s): Dr. Florent Waltz
Prof. Dr. Benjamin D. Engel

Last updated by author(s): Jun 8, 2026

Reporting Summary

Nature Portfolio wishes to improve the reproducibility of the work that we publish. This form provides structure for consistency and transparency in reporting. For further information on Nature Portfolio policies, see our Editorial Policies and the Editorial Policy Checklist.

Statistics

For all statistical analyses, confirm that the following items are present in the figure legend, table legend, main text, or Methods section.

n/a	Confirmed	
☐	☒	The exact sample size (*n*) for each experimental group/condition, given as a discrete number and unit of measurement
☐	☒	A statement on whether measurements were taken from distinct samples or whether the same sample was measured repeatedly
☒	☐	The statistical test(s) used AND whether they are one- or two-sided<br>*Only common tests should be described solely by name; describe more complex techniques in the Methods section.*
☒	☐	A description of all covariates tested
☒	☐	A description of any assumptions or corrections, such as tests of normality and adjustment for multiple comparisons
☒	☐	A full description of the statistical parameters including central tendency (e.g. means) or other basic estimates (e.g. regression coefficient) AND variation (e.g. standard deviation) or associated estimates of uncertainty (e.g. confidence intervals)
☒	☐	For null hypothesis testing, the test statistic (e.g. *F*, *t*, *r*) with confidence intervals, effect sizes, degrees of freedom and *P* value noted<br>*Give P values as exact values whenever suitable.*
☒	☐	For Bayesian analysis, information on the choice of priors and Markov chain Monte Carlo settings
☒	☐	For hierarchical and complex designs, identification of the appropriate level for tests and full reporting of outcomes
☒	☐	Estimates of effect sizes (e.g. Cohen's *d*, Pearson's *r*), indicating how they were calculated

Our web collection on statistics for biologists contains articles on many of the points above.

Software and code

Policy information about availability of computer code

Data collection	For cryo-ET: All data analysis was performed on the cryo-ET dataset deposited under EMPIAR-1183024. Briefly, tilt-series data were collected using a Titan Krios G4 transmission electron microscope operating at 300 kV equipped with a Selectris X energy filter with a slit set to 10 eV and a Falcon 4i direct electron detector.<br>For SPA cryo-EM: Data collection was performed using a 200kV Glacios and a 300kV Titan Krios electron microscope (Thermo Fisher Scientific) both equipped with Falcon4 camera using EPU (Thermo Fisher Scientific) for automated data acquisition. Data were collected at a nominal underfocus of −0.8 to −2.0µm at a magnification of ×120k for the Glacios and ×75k for the Krios, both yielding a pixel size of 0.84Å.
Data analysis	TEM Tomography 5 and EPU software (Thermo Fisher Scientific), AreTomo3, pyTOM-match-pick, RELION5, cryoSPARC v4.3, ModelAngelo, AlphaFold2, Coot v0.9, PHENIX v1.20, ChimeraX, ArtiaX, MemBrain-seg v2

For manuscripts utilizing custom algorithms or software that are central to the research but not yet described in published literature, software must be made available to editors and reviewers. We strongly encourage code deposition in a community repository (e.g. GitHub). See the Nature Portfolio guidelines for submitting code & software for further information.

Data

Policy information about availability of data

All manuscripts must include a data availability statement. This statement should provide the following information, where applicable:

- Accession codes, unique identifiers, or web links for publicly available datasets
- A description of any restrictions on data availability
- For clinical datasets or third party data, please ensure that the statement adheres to our policy

The single particle cryo-EM maps of C. reinhardtii chlororibosome have been deposited at the Electron Microscopy Data Bank (EMDB) and models on the protein data bank (PDB). For the overall single particle reconstruction, composite map EMD-56352 (PDB : 9TVU), consensus map EMD-56337 , LSU focus EMD-56338, LSU CP focus EMD-56339, LSU L7/12 focus EMD-56340, SSU body EMD-56341 and SSU head EMD-56342, SSU extension base EMD-56349 and SSU extension tip EMD-56344.
For the SPA in presence of the factor pY composite map EMD-56602 (PDB : 28LU), consensus map EMD-56560, LSU focus EMD-56563, SSU focus EMD-56564. For the SPA with the P-site tRNA composite map EMD-56567 (PDB : 28JW), consensus map EMD-56561, LSU focus EMD-56562, SSU focus EMD-56565.
For the subtomogram average of the native Chlamydomonas chlororibosome: composite map EMD-56143, consensus map EMD-56139, LSU focus EMD-56140, SSU focus EMD-56141 and SSU extension EMD-56142, and membrane-bound class EMD-56144. The raw cryo-ET data is available at the Electron Microscopy Public Image Archive (EMPIAR), accession code: EMPIAR-11830.
Polysome and membrane association code and output are available at : https://github.com/Phaips/ChloroRibo

Research involving human participants, their data, or biological material

Policy information about studies with human participants or human data. See also policy information about sex, gender (identity/presentation), and sexual orientation and race, ethnicity and racism.

Reporting on sex and gender	na
Reporting on race, ethnicity, or other socially relevant groupings	na
Population characteristics	na
Recruitment	na
Ethics oversight	na

Note that full information on the approval of the study protocol must also be provided in the manuscript.

Field-specific reporting

Please select the one below that is the best fit for your research. If you are not sure, read the appropriate sections before making your selection.

☒ Life sciences ☐ Behavioural & social sciences ☐ Ecological, evolutionary & environmental sciences

For a reference copy of the document with all sections, see nature.com/documents/nr-reporting-summary-flat.pdf

Life sciences study design

All studies must disclose on these points even when the disclosure is negative.

Sample size	For tomography, 129 tomograms were used. For SPA cryo-EM, two samples were analyzed.
Data exclusions	no data were excluded.
Replication	not applicable to tomography. For single particle analysis, purification experiments were repeat in the same conditions and always yielded the same complexes when analyzed by cryo-EM.
Randomization	Not relevant to our study. Randomisation is however internaly applied by the image processing software (RELION and cryosparc).
Blinding	not applicable

Reporting for specific materials, systems and methods

We require information from authors about some types of materials, experimental systems and methods used in many studies. Here, indicate whether each material, system or method listed is relevant to your study. If you are not sure if a list item applies to your research, read the appropriate section before selecting a response.

Materials & experimental systems

n/a	Involved in the study
☒	☐ Antibodies
☒	☐ Eukaryotic cell lines
☒	☐ Palaeontology and archaeology
☒	☐ Animals and other organisms
☒	☐ Clinical data
☒	☐ Dual use research of concern
☒	☐ Plants

Methods

n/a	Involved in the study
☒	☐ ChIP-seq
☒	☐ Flow cytometry
☒	☐ MRI-based neuroimaging

Plants

Seed stocks	na
Novel plant genotypes	na
Authentication	na
