# Supplementary material for: Chloroplast-encoded small subunit extensions reshape the Chlamydomonas chlororibosome
Source: Nat Plants. 2026 Jul 27;12(8):1612–21. doi: 10.1038/s41477-026-02361-1 (PMC13481248; doi:10.1038/s41477-026-02361-1)
Supplement: Supplementary file 3 [file 41477_2026_2361_MOESM3_ESM.pdf]

nature portfolio

Peer Review File

Chloroplast-encoded small subunit extensions reshape the Chlamydomonas chlororibosome

Corresponding Author: Dr Florent Waltz

Version 0:

Reviewer comments:

Reviewer #1

(Comments for the Author)
This manuscript describes in situ cryo-ET and cryo-EM analyzes of the structure and in situ locations of the chlororibosome in Chlamydomonas. The resolutions obtained are considered very good to excellent. The results reveal differences with even the chloroplast ribosome of spinach. A dramatic difference is an accessory arm domain of the small subunit. The results and the comparative analyses to which they contribute are important for the field. While the results are interesting to this reviewer and likely to researcher of translation in all organisms, they are entirely descriptive and all points regarding mechanism and function are speculative or published previously.

Paragraph 50. The authors claim to visualize polysomes on thylakoid membranes based on arrays. While plausible, this claim requires showing that these ribosomes are oriented in the same direction and that at last 2 arrays have lengths consistent with mRNA length, as was done in analyzes of cytoplasmic ribosomes on mitochondria in J Cell Biol (2025) 224 (4): e202407110.

Paragraph 100. Could the heterogeneity of the chlororibosomes in SPA reflect different functional classes- thylakoid bound vs free? In any case, the authors should state 1) whether both stomal and thylakoid-bound chloribosomes had the accessory arm domain and, if not, which had it, or 2) that this could not be determined.

Minor
Paragraphs 90 and 100. The authors claim that their observation of ribosomes in the various states of the elongation cycle indicated they are translationally active. Assembled ribosomes are either active, stalled in elongation (for regulation or at a lesion in the mRNA), or hibernating. Thus, ribosomes in these states could be stalled and thus not translating. Hibernating ribosomes would have a distinct structure and could be recognized here. It could be sufficient to state that most assembled chlororibosomes are translationally active, although this is also an assumption in ribo-seq profiling papers unless they include a control with lincomycin treatment to show that most ribosomes run-off their mRNA. Some revision might be appropriate.
Similarly, is it accepted to refer to the presence of variable angles of ribosomes with respect to a membrane as “movement”. While this is plausible, qualification of this term seems necessary to this reviewer.
The second to last sentence in paragraph 80 would be more meaningful if a citation were given to the special interaction between the mitochondrial ribosome and the inner membrane translocon, for example; Science. 2021 Feb 19;371(6531):846–849
Paragraph 160. The authors state that “the r-proteins uS2c, uS3c, uS4c and uS5c”… “are all encoded in the chloroplast genome”. However, uS5c is nucleus-encoded, by RPS5 as listed in supplemental table 2, Cre16.g659950. Mention of these gene locations could be removed because I don’t see the relevance. If there is relevance, then it should be stated and uS5c removed from the list.

(Remarks on code availability)
i am not qualified to assess this.

Reviewer #2

(Comments for the Author)
The manuscript by Waltz et al. combines in situ cryo-electron tomography (cryo-ET) and single-particle cryo-electron microscopy (cryo-EM) to determine structures of the Chlamydomonas reinhardtii chlororibosome. The study identifies several structural features that appear unique to these chlororibosomes. These include (i) thylakoid-associated chlororibosomes exhibit a greater degree of positional/orientational freedom, consistent with a loose association with the thylakoid translocon, in contrast to ER-bound cytosolic ribosomes or mitoribosomes, which are typically more rigidly docked; (ii) a ~5 Å subtomogram average reveals an additional density on the solvent side of the small ribosomal subunit (SSU) that extends

from the SSU body to the SSU head, termed the “arm” domain; and (iii) the arm domain is composed of extensions of conserved SSU ribosomal proteins (uS2c, uS3c, uS4c, and uS5c) along with chloroplast-specific proteins cS24 (PSRP3), cS25 (PSRP7), and cS26 (PSRP8). Notably, the arm is positioned near the mRNA entry and exit sites, suggesting its potential role in stabilizing mRNA during translation.
Overall, this is a fascinating study that provides novel insight into the architecture of the C. reinhardtii chlororibosome. The work also supports the idea that ribosomal protein extensions can evolve into distinct structural modules, potentially enabling additional regulatory functions in chlororibosome-mediated translation. That said, clarification on following few points would further strengthen the manuscript and improve clarity for a broader readership of the journal:
1. How did the authors rule out the possibility that the arm domain includes accessory proteins beyond ribosomal proteins? Is there mass spectrometry (or another compositional analysis) performed for the purified chlororibosome preparation? If so, it should be included in the paper; if not, there should be a justified reason for not having such data.
2. The arm domain is observed only in a subset of particles (~24%), and the authors attribute this to disordering during purification. Is there any correlation between ribosome ligand state and arm density? For example, is the arm density better defined in the translating/stalled state with mRNA and P-site tRNA, given the proposed role in mRNA stabilization?
3. Related to point 2, do the authors observe any change in arm conformation or density when pY (previously identified as PSRP1) occupies the mRNA channel?
4. Since the arm spans from the SSU body to the head, it would be helpful if the authors could comment on how EF-G–mediated tRNA–mRNA translocation might be influenced by this additional domain. Would it restrict the ratchet-like inter- or intra-subunit rotation of the SSU.
5. Minor: Please include a supplementary figure showing the individual fits of the arm constituents (uS2c, uS3c, uS4c, uS5c, cS24, cS25, and cS26) into their respective densities.
6. Original cryo-EM study describing structures of the mammalian mitochondria (e.g., PMID: 14532006) and related reviews (e.g., DOI: 10.1007/978-3-642-39426-3_1, and PMID: 37166629) should be cited in the introduction section.

(Remarks on code availability)

Reviewer #3

(Comments for the Author)
In this work, Waltz et al. present structures of Chloroplast ribosomes (chlororibosomes) from Chlamydomonas reinhardtii using a combination of in situ cryo-electron tomography (cryo-ET) and single-particle cryo-electron microscopy (cryo-EM). They determine structures of the chlororibosomes attached to thylakoid membranes and those free in stroma. These ribosomes were captured in various steps of translation and in a translation-inhibited state. Interestingly, they reveal that chlororibosomes from Chlamydomonas reinhardtii possess unique extensions in small subunit ribosomal proteins and chlororibosome-specific ribosomal proteins. These unique extensions form an additional ‘arm’ in the small subunit of these chlororibosomes. Using High-resolution single-particle reconstruction of isolated chlororibosomes they figured out the details of this ‘arm’domain. The analysis suggests a role in stabilizing mRNA as this domain is located around the entry and exit channels of mRNA.
The work done is of high quality and the results are very well presented. The cryo-ET and cryo-EM techniques used are solid and they reveals the structural diversity of chlororibosomes. I recommend publication of this work. I have a few questions which I would like the authors to address before the publication of this work.
Questions:
1. This work suggests that thylakoid-associated chlororibosomes are loosely tethered in contrast to other ribosomes that are held more tightly to the membrane. What are the implications of this loose association with the membrane for the chlororibosomes?
2. Regarding the absence of bTHX and chloroplast-specific r-proteins cS22 and cS23 (PSRP2 and PSRP3): Is there a possibility that these proteins were lost during the purification or these proteins are absent in Chlamydomonas reinhardtii?
3. The Chlamydomonas homolog of Alb3 is suggested to play a role in ribosomes binding to the membrane. Was this protein observed using mass spectrometry or any other method in the ribosome fraction?
4. The authors suggest that the ‘arm’ domain plays a role in ‘organizing chloroplast polysomes’. Please elaborate how this arm domain may play this role.
5. (This is not a question) In the local resolution figures (Supplementary Fig 2-4), the highest resolution is depicted in red while lowest resolution in blue. I think, the reverse scheme is generally used i.e. blue for best resolution and red for worst resolution.

(Remarks on code availability)

Decision Letter:

10th April 2026

Dear Dr Waltz,

Your Article, "Chloroplast-encoded small subunit extensions reshape the Chlamydomonas chlororibosome" has now been seen by three referees. You will see from their comments below that while they find your work of interest, some important points are raised. We are interested in the possibility of publishing your study in Nature Plants, but would like to consider your

response to these concerns in the form of a revised manuscript before we make a final decision on publication.

As you can read below, the reviewers think that the findings are interesting and important to the field, however, all reviewers have questions that should be addressed in a revised version. In our opinion the main points are:

1. Reviewer #1 states that visualization of polysomes requires support by further analysis, and wants to know whether the heterogeneity of the chlororibosomes may reflect different functional classes.
2. Reviewer #2 would like to know whether the arm domain could contain additional proteins beyond ribosomal proteins, and whether there is a correlation between ribosome ligand state and arm density. Furthermore, it should be discussed how EF-G–mediated tRNA–mRNA translocation might be influenced by the arm domain.
3. Reviewer #3 would like to know what the consequences of the more loose tethering of chlororibosomes to thylakoid membrane are, and whether Alb3 was detected in chlororibosome fractions. Finally, it should be discussed whether absence of ribosomal proteins is due to technical or biological factors.

All referees also raise some other points that should be addressed. As usual, please do not hesitate to get in touch if you would like to discuss these issues further.

We therefore invite you to revise your manuscript taking into account all reviewer and editor comments. Please highlight all changes in the manuscript text file, preferably in Microsoft Word format.

We are committed to providing a fair and constructive peer-review process. Do not hesitate to contact us if there are specific requests from the reviewers that you believe are technically impossible or unlikely to yield a meaningful outcome.

When revising your manuscript please:

* Include a "Response to referees" document detailing, point-by-point, how you addressed each referee comment. If no action was taken to address a point, you must provide a compelling argument. This response will be sent back to the referees along with the revised manuscript.

* If you have not done so already please begin to revise your manuscript so that it conforms to our Article format instructions at http://www.nature.com/nplants/info/final-submission. Refer also to any guidelines provided in this letter.

* Include a revised version of any required reporting checklist. It will be available to referees (and, potentially, statisticians) to aid in their evaluation if the manuscript goes back for peer review. A revised checklist is essential for re-review of the paper.

* Pay close attention to our href="https://www.nature.com/nature-portfolio/editorial-policies/image-integrity">Digital Image Integrity Guidelines. and to the following points. Please ensure:
-- that unprocessed scans are clearly labelled and match the gels and western blots presented in figures.
-- that control panels for gels and western blots are appropriately described as loading on sample processing controls
-- that all images in the paper are checked for duplication of panels and for splicing of gel lanes.
-- that you retain unprocessed data and metadata files after publication, ideally archiving data in perpetuity. These may be requested during the peer review and production process or after publication if any issues arise.

EXTENDED DATA FIGURES

When re-submitting your manuscript, please ensure that any supplementary figures and tables that are crucial to the manuscript's conclusions are converted into Extended Data figures and tables to increase visibility of these data. Extended Data figures and tables are online-only (present in the online PDF and full-text HTML versions of the paper), peer-reviewed display items that provide essential background to the article but are not included in the main article due to space constraints. A maximum of ten Extended Data display items (figures and tables) is permitted.

Nature Plants strongly supports public availability of data and are therefore keen that the data used in your paper is placed in an appropriate public data repository. Alternatively, if this is not possible, you may present the data as Extended Data or Supplementary Information. If data can only be shared on request, please explain why in your Data Availability Statement, and also in the correspondence with your editor. Please note that for some data types, deposition in a public repository is mandatory.

Please use the link below to submit your revised manuscript and related files:

Link Redacted

Note: This URL links to your confidential home page and associated information about manuscripts you may have submitted, or that you are reviewing for us. If you wish to forward this email to co-authors, please delete the link to your homepage.

We hope to receive your revised manuscript within two to three months, however, this is not a strict deadline. If you cannot send it within this time, please let us know. We will be happy to consider your revision so long as nothing similar has been accepted for publication at Nature Plants or published elsewhere.

We look forward to seeing the revised manuscript and thank you for the opportunity to review your work.

Sincerely,

Nature Plants is committed to improving transparency in authorship. As part of our efforts in this direction, we are now requesting that all authors identified as 'corresponding author' on published papers create and link their Open Researcher and Contributor Identifier (ORCID) with their account on the Manuscript Tracking System (MTS), prior to acceptance. This applies to primary research papers only. ORCID helps the scientific community achieve unambiguous attribution of all scholarly contributions. You can create and link your ORCID from the home page of the MTS by clicking on 'Modify my Springer Nature account'. For more information please visit please visit www.springernature.com/orcid.

Please do not hesitate to contact me if you have any questions or would like to discuss these revisions further.

****

Reviewers' Comments:

Reviewer #1 (Comments for the Author):

This manuscript describes in situ cryo-ET and cryo-EM analyzes of the structure and in situ locations of the chlororibosome in Chlamydomonas. The resolutions obtained are considered very good to excellent. The results reveal differences with even the chloroplast ribosome of spinach. A dramatic difference is an accessory arm domain of the small subunit. The results and the comparative analyses to which they contribute are important for the field. While the results are interesting to this reviewer and likely to researcher of translation in all organisms, they are entirely descriptive and all points regarding mechanism and function are speculative or published previously.

Paragraph 50. The authors claim to visualize polysomes on thylakoid membranes based on arrays. While plausible, this claim requires showing that these ribosomes are oriented in the same direction and that at last 2 arrays have lengths consistent with mRNA length, as was done in analyzes of cytoplasmic ribosomes on mitochondria in J Cell Biol (2025) 224 (4): e202407110.

Paragraph 100. Could the heterogeneity of the chlororibosomes in SPA reflect different functional classes- thylakoid bound vs free? In any case, the authors should state 1) whether both stomal and thylakoid-bound chloribosomes had the accessory arm domain and, if not, which had it, or 2) that this could not be determined.

Minor
Paragraphs 90 and 100. The authors claim that their observation of ribosomes in the various states of the elongation cycle indicated they are translationally active. Assembled ribosomes are either active, stalled in elongation (for regulation or at a lesion in the mRNA), or hibernating. Thus, ribosomes in these states could be stalled and thus not translating. Hibernating ribosomes would have a distinct structure and could be recognized here. It could be sufficient to state that most assembled chlororibosomes are translationally active, although this is also an assumption in ribo-seq profiling papers unless they include a control with lincomycin treatment to show that most ribosomes run-off their mRNA. Some revision might be appropriate.
Similarly, is it accepted to refer to the presence of variable angles of ribosomes with respect to a membrane as “movement”. While this is plausible, qualification of this term seems necessary to this reviewer.
The second to last sentence in paragraph 80 would be more meaningful if a citation were given to the special interaction between the mitochondrial ribosome and the inner membrane translocon, for example; Science. 2021 Feb 19;371(6531):846–849
Paragraph 160. The authors state that “the r-proteins uS2c, uS3c, uS4c and uS5c”… “are all encoded in the chloroplast genome”. However, uS5c is nucleus-encoded, by RPS5 as listed in supplemental table 2, Cre16.g659950. Mention of these gene locations could be removed because I don’t see the relevance. If there is relevance, then it should be stated and uS5c removed from the list.

Reviewer #2 (Comments for the Author):

The manuscript by Waltz et al. combines in situ cryo-electron tomography (cryo-ET) and single-particle cryo-electron microscopy (cryo-EM) to determine structures of the Chlamydomonas reinhardtii chlororibosome. The study identifies several structural features that appear unique to these chlororibosomes. These include (i) thylakoid-associated chlororibosomes exhibit a greater degree of positional/orientational freedom, consistent with a loose association with the thylakoid translocon, in contrast to ER-bound cytosolic ribosomes or mitoribosomes, which are typically more rigidly docked; (ii) a ~5 Å subtomogram average reveals an additional density on the solvent side of the small ribosomal subunit (SSU) that extends from the SSU body to the SSU head, termed the “arm” domain; and (iii) the arm domain is composed of extensions of conserved SSU ribosomal proteins (uS2c, uS3c, uS4c, and uS5c) along with chloroplast-specific proteins cS24 (PSRP3), cS25 (PSRP7), and cS26 (PSRP8). Notably, the arm is positioned near the mRNA entry and exit sites, suggesting its potential role in stabilizing mRNA during translation.
Overall, this is a fascinating study that provides novel insight into the architecture of the C. reinhardtii chlororibosome. The

work also supports the idea that ribosomal protein extensions can evolve into distinct structural modules, potentially enabling additional regulatory functions in chlororibosome-mediated translation. That said, clarification on following few points would further strengthen the manuscript and improve clarity for a broader readership of the journal:
1. How did the authors rule out the possibility that the arm domain includes accessory proteins beyond ribosomal proteins? Is there mass spectrometry (or another compositional analysis) performed for the purified chlororibosome preparation? If so, it should be included in the paper; if not, there should be a justified reason for not having such data.
2. The arm domain is observed only in a subset of particles (~24%), and the authors attribute this to disordering during purification. Is there any correlation between ribosome ligand state and arm density? For example, is the arm density better defined in the translating/stalled state with mRNA and P-site tRNA, given the proposed role in mRNA stabilization?
3. Related to point 2, do the authors observe any change in arm conformation or density when pY (previously identified as PSRP1) occupies the mRNA channel?
4. Since the arm spans from the SSU body to the head, it would be helpful if the authors could comment on how EF-G–mediated tRNA–mRNA translocation might be influenced by this additional domain. Would it restrict the ratchet-like inter- or intra-subunit rotation of the SSU.
5. Minor: Please include a supplementary figure showing the individual fits of the arm constituents (uS2c, uS3c, uS4c, uS5c, cS24, cS25, and cS26) into their respective densities.
6. Original cryo-EM study describing structures of the mammalian mitochondria (e.g., PMID: 14532006) and related reviews (e.g., DOI: 10.1007/978-3-642-39426-3_1, and PMID: 37166629) should be cited in the introduction section.

Reviewer #3 (Comments for the Author):

In this work, Waltz et al. present structures of Chloroplast ribosomes (chlororibosomes) from Chlamydomonas reinhardtii using a combination of in situ cryo-electron tomography (cryo-ET) and single-particle cryo-electron microscopy (cryo-EM). They determine structures of the chlororibosomes attached to thylakoid membranes and those free in stroma. These ribosomes were captured in various steps of translation and in a translation-inhibited state. Interestingly, they reveal that chlororibosomes from Chlamydomonas reinhardtii possess unique extensions in small subunit ribosomal proteins and chlororibosome-specific ribosomal proteins. These unique extensions form an additional ‘arm’ in the small subunit of these chlororibosomes. Using High-resolution single-particle reconstruction of isolated chlororibosomes they figured out the details of this ‘arm’domain. The analysis suggests a role in stabilizing mRNA as this domain is located around the entry and exit channels of mRNA.
The work done is of high quality and the results are very well presented. The cryo-ET and cryo-EM techniques used are solid and they reveals the structural diversity of chlororibosomes. I recommend publication of this work. I have a few questions which I would like the authors to address before the publication of this work.
Questions:
1. This work suggests that thylakoid-associated chlororibosomes are loosely tethered in contrast to other ribosomes that are held more tightly to the membrane. What are the implications of this loose association with the membrane for the chlororibosomes?
2. Regarding the absence of bTHX and chloroplast-specific r-proteins cS22 and cS23 (PSRP2 and PSRP3): Is there a possibility that these proteins were lost during the purification or these proteins are absent in Chlamydomonas reinhardtii?
3. The Chlamydomonas homolog of Alb3 is suggested to play a role in ribosomes binding to the membrane. Was this protein observed using mass spectrometry or any other method in the ribosome fraction?
4. The authors suggest that the ‘arm’ domain plays a role in ‘organizing chloroplast polysomes’. Please elaborate how this arm domain may play this role.
5. (This is not a question) In the local resolution figures (Supplementary Fig 2-4), the highest resolution is depicted in red while lowest resolution in blue. I think, the reverse scheme is generally used i.e. blue for best resolution and red for worst resolution.

********************END********************

Version 1:

Reviewer comments:

Reviewer #1

(Comments for the Author)
The authors have addressed my points satisfactorily. I have a two minor suggestions.
Line 72 “Thylakoid-associated chloro-ribosomes are loosely tethered yet actively translating in situ”. Here, “loosely” implies “weakly”, high Kd. The authors might consider “flexibly tethered” or other wording to reflect flexibility or distance from the membrane, rather than affinity for it.
Chlamydomonas should be italicized. The rule that model organism names are not italicized seems to have changed.

(Remarks on code availability)

Reviewer #2

(Comments for the Author)
I am satisfied with authors' most responses in the revised version and find the manuscript significantly improved.

(Remarks on code availability)

Reviewer #3

(Comments for the Author)
In the revised submission, the authors have satisfactorily addressed all the concerns raised. I congratulate the authors on this exciting finding in chloroplast ribosomes.

(Remarks on code availability)

Decision Letter:

Our ref: NPLANTS-260222321A

1st June 2026

Dear Dr. Waltz,

Thank you for submitting your revised manuscript "Chloroplast-encoded small subunit extensions reshape the Chlamydomonas chlororibosome" (NPLANTS-260222321A). It has now been seen by the original referees and their comments are below. The reviewers find that the paper has improved in revision, and therefore we'll be happy in principle to publish it in Nature Plants, pending minor revisions to satisfy the referees' final requests and to comply with our editorial and formatting guidelines.

We are now performing detailed checks on your paper and will send you a checklist detailing our editorial and formatting requirements in about a week. Please do not upload the final materials and make any revisions until you receive this additional information from us.

Thank you again for your interest in Nature Plants Please do not hesitate to contact me if you have any questions.

Sincerely,

Reviewer #1 (Comments for the Author):

The authors have addressed my points satisfactorily. I have a two minor suggestions.
Line 72 “Thylakoid-associated chloro-ribosomes are loosely tethered yet actively translating in situ”. Here, “loosely” implies “weakly”, high Kd. The authors might consider “flexibly tethered” or other wording to reflect flexibility or distance from the membrane, rather than affinity for it.
Chlamydomonas should be italicized. The rule that model organism names are not italicized seems to have changed.

Reviewer #2 (Comments for the Author):

I am satisfied with authors' most responses in the revised version and find the manuscript significantly improved.

Reviewer #3 (Comments for the Author):

In the revised submission, the authors have satisfactorily addressed all the concerns raised. I congratulate the authors on this exciting finding in chloroplast ribosomes.

Version 2:

Decision Letter:

8th July 2026

Dear Dr Waltz,

We are pleased to inform you that your Article entitled "Chloroplast-encoded small subunit extensions reshape the Chlamydomonas chlororibosome", has now been accepted for publication in Nature Plants.

Over the next few weeks, your paper will be copyedited to ensure that it conforms to Nature Plants style. We look particularly carefully at the titles of all papers to ensure that they are relatively brief and understandable.

Once your paper is typeset, you will receive an email with a link to choose the appropriate publishing options for your paper and our Author Services team will be in touch regarding any additional information that may be required.

After the grant of rights is completed, you will receive a link to your electronic proof via email with a request to make any corrections within 48 hours. If, when you receive your proof, you cannot meet this deadline, please inform us at rjsproduction@springernature.com immediately.

You will not receive your proofs until the publishing agreement has been received through our system.

You may wish to make your media relations office aware of your accepted publication, in case they consider it appropriate to organize some internal or external publicity. Once your paper has been scheduled you will receive an email confirming the publication details. This is normally 3-4 working days in advance of publication. If you need additional notice of the date and time of publication, please let the production team know when you receive the proof of your article to ensure there is sufficient time to coordinate. Further information on our embargo policies can be found here: https://www.nature.com/authors/policies/embargo.html

Due to the importance of these deadlines, we ask you please us know now whether you will be difficult to contact over the next month. If this is the case, we ask you provide us with the contact information (email, phone and fax) of someone who will be able to check the proofs on your behalf, and who will be available to address any last-minute problems.

Acceptance of your manuscript is conditional on all authors' agreement with our publication policies (see http://www.nature.com/authors/policies/index.html). In particular your manuscript must not be published elsewhere.

If you have any questions about our publishing options, costs, Open Access requirements, or our legal forms, please contact ASJournals@springernature.com

Authors may need to take specific actions to achieve compliance with funder and institutional open access mandates. If your research is supported by a funder that requires immediate open access (e.g. according to Plan S principles or the NIH public access policy) then you should select the gold OA route, and we will direct you to the compliant route where possible. Because authors warrant under our subscription licensing terms that they haven’t committed to licensing any version of their article under a licence inconsistent with the terms of our agreement – including the applicable embargo period – publication under the subscription model isn’t suitable for authors whose funders require no embargo.

An online order form for reprints of your paper is available at https://www.nature.com/reprints/author-reprints.html. All co-authors, authors' institutions and authors' funding agencies can order reprints using the form appropriate to their geographical region.

We welcome the submission of potential cover material (including a short caption of around 40 words) related to your manuscript; suggestions should be sent to Nature Plants as electronic files (the image should be 300 dpi at 210 x 297 mm in either TIFF or JPEG format). Please note that such pictures should be selected more for their aesthetic appeal than for their scientific content, and that colour images work better than black and white or grayscale images. Please include a written description of your image and how it was created along with your image files. Please do not try to design a cover with the Nature Plants logo etc., and please do not submit composites of images related to your work. I am sure you will understand that we cannot make any promise as to whether any of your suggestions might be selected for the cover of the journal.

You can now use a single sign-on for all your accounts, view the status of all your manuscript submissions and reviews, access usage statistics for your published articles and download a record of your refereeing activity for the Nature journals.

To assist our authors in disseminating their research to the broader community, our SharedIt initiative provides you with a unique shareable link that will allow anyone (with or without a subscription) to read the published article. Recipients of the link with a subscription will also be able to download and print the PDF.

As soon as your article is published, you will receive an automated email with your shareable link.

With kind regards,

P.S. Click on the following link if you would like to recommend Nature Plants to your librarian
http://www.nature.com/subscriptions/recommend.html#forms

** Visit the Springer Nature Editorial and Publishing website at www.springernature.com/editorial-and-publishing-jobs for more information about our career opportunities. If you have any questions please click here.**

Open Access This Peer Review File is licensed under a Creative Commons Attribution 4.0 International License, which permits use, sharing, adaptation, distribution and reproduction in any medium or format, as long as you give appropriate credit to the original author(s) and the source, provide a link to the Creative Commons license, and indicate if changes were made.
In cases where reviewers are anonymous, credit should be given to 'Anonymous Referee' and the source.
The images or other third party material in this Peer Review File are included in the article's Creative Commons license, unless indicated otherwise in a credit line to the material. If material is not included in the article's Creative Commons license and your intended use is not permitted by statutory regulation or exceeds the permitted use, you will need to obtain permission directly from the copyright holder.
To view a copy of this license, visit https://creativecommons.org/licenses/by/4.0/

Detailed response to reviewers:

Reviewer #1

We thank Reviewer 1 for their positive appreciation of the work.

Paragraph 50. The authors claim to visualize polysomes on thylakoid membranes based on arrays. While plausible, this claim requires showing that these ribosomes are oriented in the same direction and that at last 2 arrays have lengths consistent with mRNA length, as was done in analyzes of cytoplasmic ribosomes on mitochondria in J Cell Biol (2025) 224 (4): e202407110.

We have now prepared a new supplementary figure 2 that includes a complete polysome analysis showing that these are indeed polysomes.

Additionally, we checked the theoretical maximum polysome lengths, considering the 69 transcripts of the Chlamydomonas chloroplast genome. Counting membrane proteins only, transcripts psaA and psaB (coding for Photosystem I subunits) are the largest proteins, followed by psbB (Photosystem II subunit) which are respectively 751, 735 and 508 amino acids. Considering the CDS only, this corresponds to 2253, 2205 and 1524 nucleotides. If we consider an average ribosome footprint of 30 nt, this would correspond to a maximum of 75 ribosomes. If we consider a larger footprint of 50 nt, or a looser polysome (more spacing between the ribosomes), this would correspond to a polysome of 45 ribosomes, which is well beyond to the maximum length of 14 we observed, confirming that our findings are physically plausible.

Paragraph 100. Could the heterogeneity of the chlororibosomes in SPA reflect different functional classes- thylakoid bound vs free? In any case, the authors should state 1) whether both stomal and thylakoid-bound chloribosomes had the accessory arm domain and, if not, which had it, or 2) that this could not be determined.

If the reviewer is referring to the absence/presence of the arm domain, we believe the structural heterogeneity observed in SPA is purely due to the purification procedure. The arm domain has 100% occupancy in situ, regardless of whether ribosomes are free or thylakoid-bound. Since this has been asked also by the other reviewers, we clarified the occupancy of the arm domain in situ in the text.

Additionally, even though not the focus of the work, we have observed the arm domain in different conditions with the same 100% occupancy in a different study (https://www.biorxiv.org/content/10.64898/2026.01.27.701992v1) as well as unpublished work.

Minor

Paragraphs 90 and 100. The authors claim that their observation of ribosomes in the various states of the elongation cycle indicated they are translationally active. Assembled ribosomes are either active, stalled in elongation (for regulation or at a lesion in the mRNA), or hibernating. Thus, ribosomes in these states could be stalled and thus not translating. Hibernating ribosomes would have a distinct structure and could be recognized here. It could be sufficient to state that most assembled chlororibosomes are translationally active, although this is also an assumption in ribo-seq profiling papers unless they include a control with lincomycin treatment to show that most ribosomes run-off their mRNA. Some revision might be appropriate.

We agree that the presence of tRNA occupancy and elongation factors, while characteristic of the elongation cycle, does not strictly rule out the possibility of ribosomes being stalled.

In response to this comment, we have revised the text in Paragraphs 90 and 100 to adopt a more conservative interpretation. We now state that our observations are "consistent with" active translation

and acknowledge that these structures could also represent stalled states, aligning our terminology with the standard assumptions used in the ribo-seq field.

Regarding the state of hibernation: while we cannot entirely exclude the presence of the plastid-specific hibernation factor (PY) as a potential low-occupancy substate in situ, we did not observe the characteristic disome structures (100S) often associated with certain hibernation pathways. However, to remain precise, we have adjusted the discussion to clarify that while hibernation is not structurally apparent in our averages, the distinction between active and stalled elongation remains an assumption based on the diversity of the observed states.

Similarly, is it accepted to refer to the presence of variable angles of ribosomes with respect to a membrane as "movement". While this is plausible, qualification of this term seems necessary to this reviewer.

Thank you. "Movement" is indeed not the appropriate word, as the variability in orientation appears to be stochastic. We replaced the word "movement" in the text by "orientation" or "orientation variability".

The second to last sentence in paragraph 80 would be more meaningful if a citation were given to the special interaction between the mitochondrial ribosome and the inner membrane translocon, for example; Science. 2021 Feb 19;371(6531):846–849

This is now added.

Paragraph 160. The authors state that "the r-proteins uS2c, uS3c, uS4c and uS5c"… "are all encoded in the chloroplast genome". However, uS5c is nucleus-encoded, by RPS5 as listed in supplemental table 2, Cre16.g659950. Mention of these gene locations could be removed because I don't see the relevance. If there is relevance, then it should be stated and uS5c removed from the list.

We clarified the sentence.

<u>Reviewer #2</u>

We appreciate Reviewer 2's supportive evaluation of our research.

1. How did the authors rule out the possibility that the arm domain includes accessory proteins beyond ribosomal proteins? Is there mass spectrometry (or another compositional analysis) performed for the purified chlororibosome preparation? If so, it should be included in the paper; if not, there should be a justified reason for not having such data.

Concerning potential additional proteins in the arm domain, we include a new supplementary figure 7, which presents the models of the extensions and specific r-proteins in their cryo-EM density, showing that the model is complete and that no density was left empty. Of course, it is quite possible that other proteins bind transiently and sub-stoichiometrically to the arm domain, such as trans-acting factors, but these would not be considered core ribosomal subunits.

To follow this idea, we also present a difference map against the model for both SPA and STA. Red areas show the unmodeled densities. As expected, the only unmodeled densities correspond to variable factors (mRNAs or translation factors and tRNAs) and unstable core components of the ribosome such as the L1 stalk or the N-ter part of uS2c (which was already presented in its own supplementary figure).

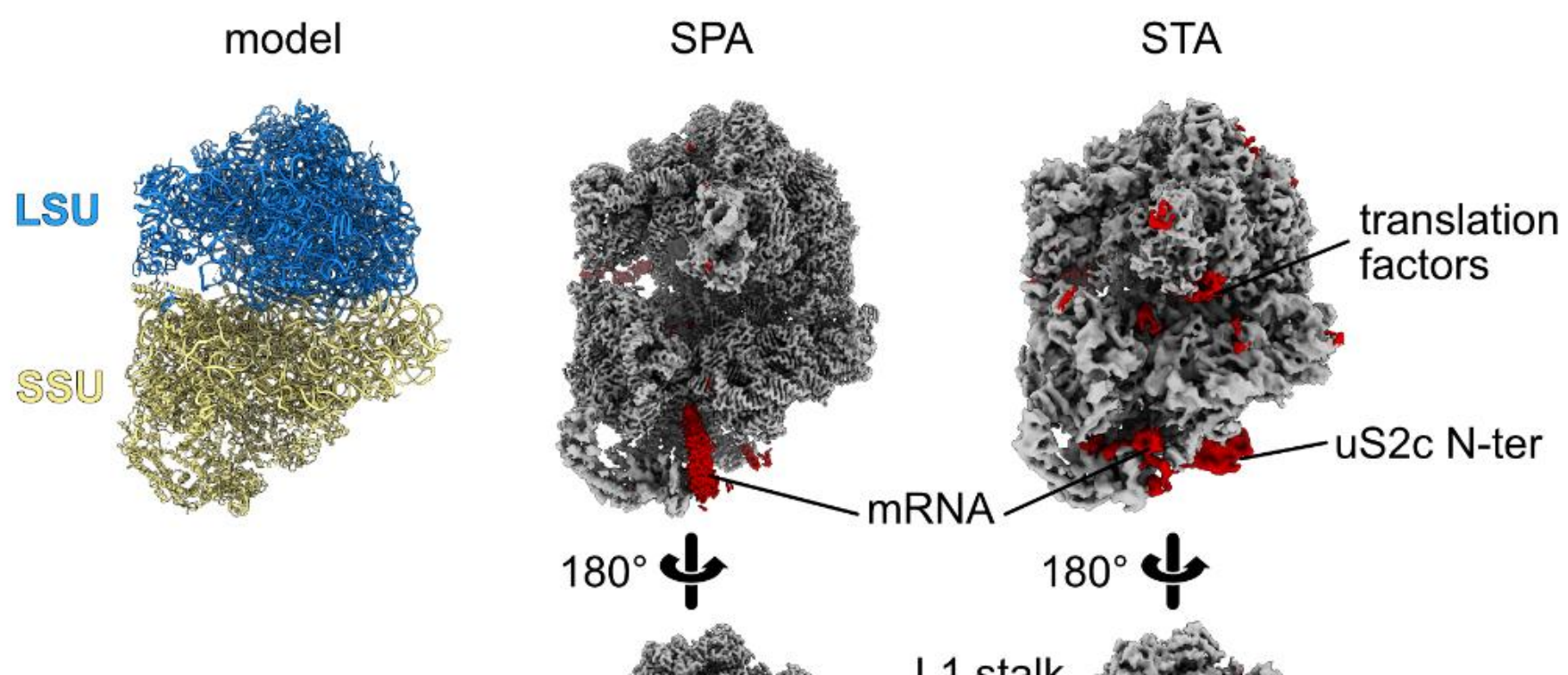

Concerning mass spectrometry, extensive analyses on the interactome of the SSU and LSU have already been carried out previously, and are publicly available (Westrich et al 2020, already cited) and were used to double check the identification of the proteins. Considering that, we didn't feel the need to repeat the same experiments.

2. The arm domain is observed only in a subset of particles (~24%), and the authors attribute this to disordering during purification. Is there any correlation between ribosome ligand state and arm density? For example, is the arm density better defined in the translating/stalled state with mRNA and P-site tRNA, given the proposed role in mRNA stabilization?

In situ, all chlororibosomes have the arm domain, in a similar conformation. In the purified sample, whether in presence of tRNA or pY factor, a similar fraction of the ribosomes have a stabilized:destabilized arm domain. A correlation between the arm density and ribosome state is one of the hypotheses we originally tested (by purifying the ribosomes in presence of chloramphenicol), but this is not what we observed.

3. Related to point 2, do the authors observe any change in arm conformation or density when pY (previously identified as PSRP1) occupies the mRNA channel?

The arm domain conformation appears to be identical in both cases.

4. Since the arm spans from the SSU body to the head, it would be helpful if the authors could comment on how EF-G–mediated tRNA–mRNA translocation might be influenced by this additional domain. Would it restrict the ratchet-like inter- or intra-subunit rotation of the SSU.

This is an interesting question. We attempted classification using an SSU-focused mask on an LSU-refined set of particles. This is quite challenging with the number of particles we have, however we managed to resolve inter-subunit (SSU compared to LSU) rotation and slight intra-subunit rotation (head relative to body). The rotation appears indeed to be restricted compared to classical bacterial ribosome, but again, the limited amount of particle makes a definitive conclusion difficult. We observed that the arm domain follows the overall movement of the subunits, likely facilitated by the hinge-like structure of the uS2c and uS5c extensions, which still allows for motion.

Here is the output of the classification, view on the SSU head, with each map is colored differently.

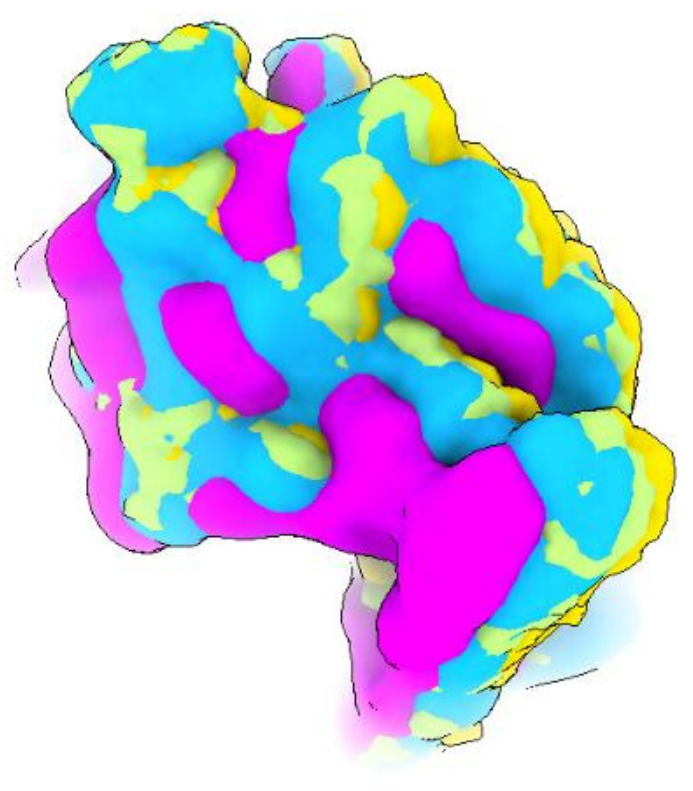

5. Minor: Please include a supplementary figure showing the individual fits of the arm constituents (uS2c, uS3c, uS4c, uS5c, cS24, cS25, and cS26) into their respective densities.

This is now presented in a new supplementary figure 7.

6. Original cryo-EM study describing structures of the mammalian mitochondria (e.g., PMID: 14532006) and related reviews (e.g., DOI: 10.1007/978-3-642-39426-3_1, and PMID: 37166629) should be cited in the introduction section.

These are now added.

Reviewer #3

We are grateful to Reviewer 3 for their positive feedback on this work.

1. This work suggests that thylakoid-associated chlororibosomes are loosely tethered in contrast to other ribosomes that are held more tightly to the membrane. What are the implications of this loose association with the membrane for the chlororibosomes?

We can only speculate. If we think about other organellar systems, in mitochondria, where the association with the membrane has been well characterized (in Chlamydomonas, animals, and yeast), the association is quite rigid. However, in these systems, the entire mitochondrial-encoded proteome consists of membrane proteins (except for one putative protein in Chlamydomonas, and one protein in yeast). Therefore, it makes sense that these systems evolved towards an almost exclusive membrane association.

However, this is not the case in the chloroplast, as half of the chloroplast-encoded proteome is soluble. This could explain why the selection towards stable membrane association was not as strong as in mitochondria. We could maybe expect that in a mitochondrial system where a large portion of the mitochondrial-encoded proteome is soluble (e.g., in land plants or in protists), a similarly loose interaction of the mitoribosomes with the translocase could exist. However, this has not yet been studied.

A related explanation could be that more loosely associated ribosomes could more easily switch from soluble to membrane-bound (and the other way around). This could be important for fast adaptation to different light conditions where repair and/or replacement of photosystem subunits and newly synthetized proteins must happen quickly.

A short statement has been added in the main text.

2. Regarding the absence of bTHX and chloroplast-specific r-proteins cS22 and cS23 (PSRP2 and PSRP3): Is there a possibility that these proteins were lost during the purification or these proteins are absent in Chlamydomonas reinhardtii?

In the case of the SPA, it is possible that factors could have been lost during purification. It should also be noted that PSRP2 and PSRP3 were not well resolved in the original spinach SPA papers. However, as shown by supplementary figure 11, comparing the SPA and STA maps, these proteins are also absent

in the *in situ* average, which would be seen at the resolution we obtained, excluding all possibility that these proteins are missing due to a purification artefact.

3. The Chlamydomonas homolog of Alb3 is suggested to play a role in ribosomes binding to the membrane. Was this protein observed using mass spectrometry or any other method in the ribosome fraction?

This protein is indeed present in Westrich et al 2020, as the only translocase identified co-immunoprecipitating with Chlamydomonas chlororibosomes. It also behaves in a similar fashion to other components of the chlororibosome. This is now additionally added to the text.

Here's the supplementary table from Westrich et al recapitulating the co-IP experiments. Red lines indicate lines and columns that were hidden for space constraints, Alb3 is highlighted in yellow and indicated with a red arrow.

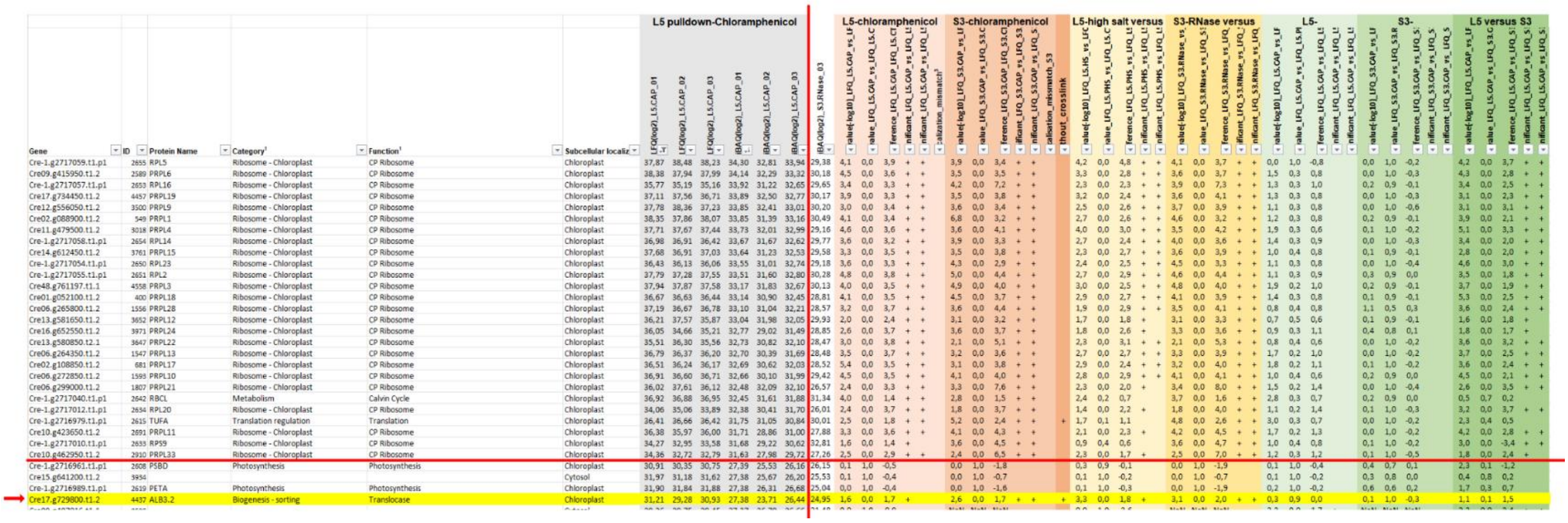

4. The authors suggest that the 'arm' domain plays a role in 'organizing chloroplast polysomes'. Please elaborate how this arm domain may play this role.

Considering that we observe that the base of the arm domain is probably involved in mRNA channeling, this would indirectly have a role in polysome formation. We have toned down our wording.

5. (This is not a question) In the local resolution figures (Supplementary Fig 2-4), the highest resolution is depicted in red while lowest resolution in blue. I think, the reverse scheme is generally used i.e. blue for best resolution and red for worst resolution.

A wide variety of color palettes have been used in the field, from blue to red to rainbow to, in some case, pink to green. There is no clear standard, as long as the scale is clearly presented and not misleading (which we hope is not the case here).

Detailed response to reviewers:

Reviewer #1:
Comments for the Author:
The authors have addressed my points satisfactorily. I have a two minor suggestions.
Line 72 "Thylakoid-associated chloro-ribosomes are loosely tethered yet actively translating in situ". Here, "loosely" implies "weakly", high Kd. The authors might consider "flexibly tethered" or other wording to reflect flexibility or distance from the membrane, rather than affinity for it.
Chlamydomonas should be italicized. The rule that model organism names are not italicized seems to have changed.

We thank reviewers 1 for their positive appreciation of the work and their constructive comments. "Flexibly" is now used in the text

Reviewer #2:
Comments for the Author:
I am satisfied with authors' most responses in the revised version and find the manuscript significantly improved.

Reviewer #3:
Comments for the Author:
In the revised submission, the authors have satisfactorily addressed all the concerns raised. I congratulate the authors on this exciting finding in chloroplast ribosomes.

We thank reviewers 2 and 3 for their positive appreciation of the work and their constructive comments.
